# Decoding the Brain's Surface to Track Deeper Activity

**DOI:** 10.3389/fnimg.2022.815778

**Published:** 2022-03-17

**Authors:** Mark L. Tenzer, Jonathan M. Lisinski, Stephen M. LaConte

**Affiliations:** ^1^Fralin Biomedical Research Institute at VTC, Virginia Tech, Roanoke, VA, United States; ^2^Department of Biomedical Engineering and Mechanics, Virginia Tech, Blacksburg, VA, United States

**Keywords:** support vector machine, resting state connectivity, functional magnetic resonance imaging, multimodal, cerebral cortex

## Abstract

Neural activity can be readily and non-invasively recorded from the scalp using electromagnetic and optical signals, but unfortunately all scalp-based techniques have depth-dependent sensitivities. We hypothesize, though, that the cortex's connectivity with the rest of the brain could serve to construct proxy signals of deeper brain activity. For example, functional magnetic resonance imaging (fMRI)-derived models that link surface connectivity to deeper regions could subsequently extend the depth capabilities of other modalities. Thus, as a first step toward this goal, this study examines whether or not surface-limited support vector regression of resting-state fMRI can indeed track deeper regions and distributed networks in independent data. Our results demonstrate that depth-limited fMRI signals can in fact be calibrated to report ongoing activity of deeper brain structures. Although much future work remains to be done, the present study suggests that scalp recordings have the potential to ultimately overcome their intrinsic physical limitations by utilizing the multivariate information exchanged between the surface and the rest of the brain.

## 1. Introduction

Every neuroimaging method has intrinsic physical limitations. But even once the technology is sufficiently advanced to harness the theoretical physical capabilities of a given approach, there is often room for sometimes astounding additional methodological and algorithmic innovation. As an example, today's functional magnetic resonance imaging (fMRI) has benefited from major transformations that surpassed the perceived physical limits in terms of both resolution and acquisition speed. Specifically, MRI exceeds the diffraction limits of 43 MHz/T resolution (an approximate field-dependent wavelength of 2 m·T) by multiple orders of magnitude thanks to the insights of Paul Lauterbur that magnetic field gradients can be used to spatially encode the NMR signal and, in addition, that algorithmic approaches can be used to reconstruct images from such data (Lauterbur, 1973). The use of magnetic gradients, though, makes MRI slow. Nonetheless major speed gains have been realized over the years by repeatedly looking beyond the physical limits of the current conventions. Echoplanar imaging (Mansfield, 1977), in-plane parallel imaging (Sodickson and Manning, 1997; Pruessmann et al., 1999; Griswold et al., 2002), and simultaneous multi-slice acquisitions (Larkman et al., 2001; Nunes et al., 2006; Feinberg et al., 2010; Moeller et al., 2010) have all culminated to exceed previous “speed limits” by orders of magnitude and to enable sub-second sampling rates.

Although fMRI is a unique and particularly successful example, one general approach for circumventing physical limitations has been to look to multimodal imaging to merge two or more techniques with complementary capabilities. In human studies, common modalities for functional measurements include magnetoencephalography (MEG), electroencephalography (EEG), positron emission tomography (PET), functional near-infrared spectroscopy (fNIRS), and fMRI. Multimodal examples include combining structural and functional imaging (e.g., using anatomical MRI to constrain source localization in EEG Pascual-Marqui, 1999; Fiederer et al., 2016), as well as combining shared primary physiological measures (e.g., obtaining both spatial and temporal resolution from fMRI and EEG or MEG (Menon et al., 1997; Dale et al., 2000). The most common motivation for employing multimodal techniques is to simultaneously capture the highest possible level of both temporal and spatial resolution (Meyer-Lindenberg, 2010). *Depth of measurement*, however, is a related physical limitation of scalp-based modalities that has been largely overlooked in the multimodal literature.

**Figure 1:**
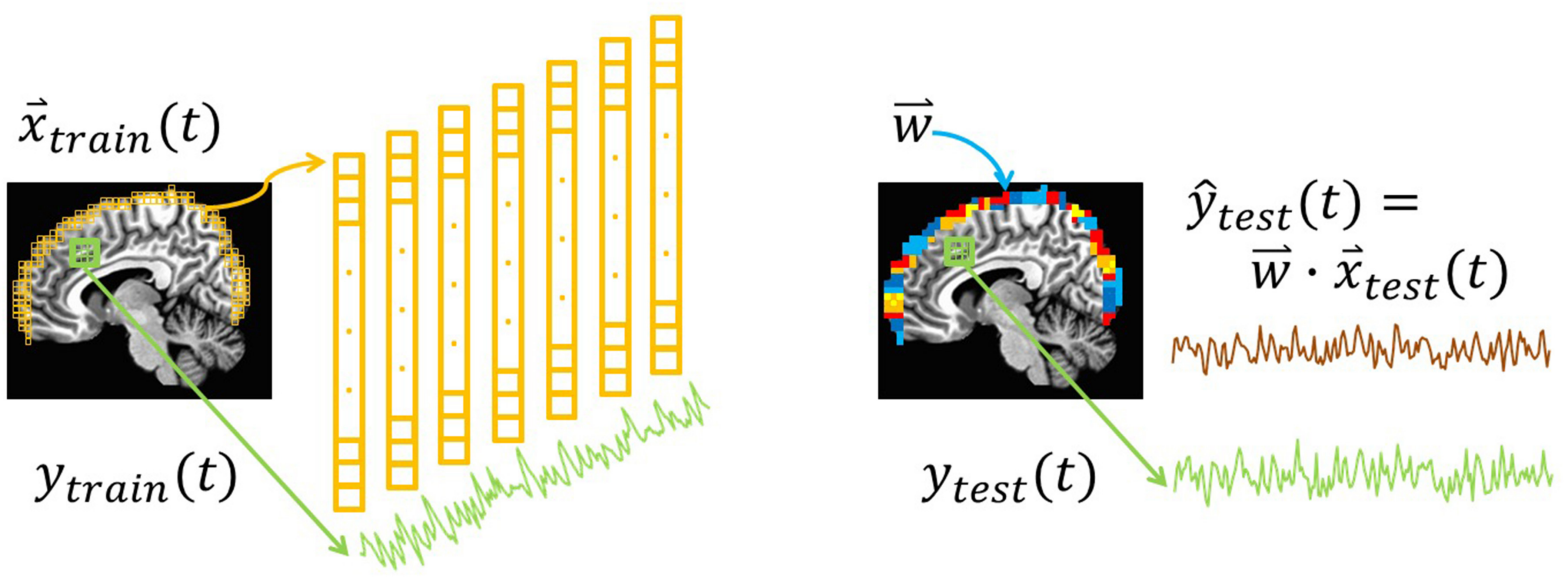
Illustration of the surface decoding approach. Left: At each point in time, voxels from the surface of the brain (yellow) are extracted. Their intensities comprise the training features, ${\vec{x}}_{train}\left(t\right)$. In addition, training labels, *y*_*train*_(*t*), are extracted from the average of voxels in a network or region of interest (green). (Not illustrated: Induction is used to train a regression model, $\vec{w}$, that relates ${\vec{x}}_{train}\left(t\right)$ to *y*_*train*_(*t*)). Right: In a new dataset, ${\vec{x}}_{test}\left(t\right)$ and *y*_*test*_(*t*) are extracted. Combining each testing vector with the regression weights $\vec{w}$ yields the predicted testing label ŷ_*test*_ (brown). In this study, the correlation between the predictions, ŷ_*test*_(*t*), and the true values, *y*_*test*_(*t*), is defined as the prediction accuracy.

To simulate approaches to bypass physical limitations in measurement depth, we used resting-state fMRI data to examine whether surface activity could serve as a proxy for deeper brain signals. The crux of this idea is the hypothesis that the multivariate connectivity of the brain “transmits” enough information to the surface of cortex to enable decoding of target signals from interior regions and networks. This study tests that basic premise by examining whether whole-brain measurements could be used to calibrate surface-restricted ones. The general concept of training a surface-limited model to track a deeper target signal is illustrated in Figure 1. Namely, a training step produces a model comprised of multivariate weight vectors that can subsequently track the target signal using only recordings from the surface. Arguably, this fMRI-based initial demonstration of feasibility would be most easily translatable to a hemodynamic measure, like fNIRS. Nevertheless, future studies will be needed to evaluate strategies for fMRI to calibrate surface modalities like EEG, fNIRS, and MEG. To fully implement a multimodal strategy around this idea will ultimately require multiple steps. Perhaps the most challenging of which would be to evaluate forward and reverse transfer functions between fMRI and the other modalities (e.g., EEG's correspondence between hemodynamic responses; Sato et al., 2010).

Currently, it is not known if even surface fMRI signals can reconstruct deeper fMRI signals. Thus, this study aimed to demonstrate that such an approach can accurately track such localized and distributed signals. Our primary question was “Can we use the surface of the brain to track distributed networks and anatomical regions?” This was examined through cross-validation on a thirteen-participant data set under two conditions i) a fixed-depth surface mask, and ii) a sparse, low resolution mask of strategic surface locations that was specific for each of the 16 target signals. The sparse masks were generated with independent data from 99 participants, which additionally enabled us to explore our secondary question “Can we map the surface connectivity that enables decoding?” Finally, we examined the reproducibility of our results by repeating our cross-validation estimates in 83 participants from an archival data set (openneuro.org; Power et al., 2017). Together, our findings suggest that surface techniques could enjoy a fundamental advancement by overcoming their intrinsic physical limitations through the use of machine learning models that capture whole brain functional connectivity.

## 2. Materials and Methods

### Participants and Data Collection

A group of 13 participants (eight females, mean age 26 years) was used to estimate cross-validated prediction accuracies. A second, larger group of 99 participants (48 females; mean age 25 years) was formed from six ongoing protocols in the lab and used to construct group-level support vector regression (SVR) maps and volumetrically-subdivided feature masks. All participants gave informed consent, and the study was done in accordance with the Institutional Review Board of Virginia Tech.

Structural and functional brain data were acquired on a 3 Tesla whole-body scanner (Siemens Trio). A three-dimensional magnetization prepared rapid acquisition gradient echo (MPRAGE) pulse sequence (Mugler and Brookeman, 1990) was used to collect T1-weighted anatomical volumes (resolution = 1 × 1 × 1 mm^3^; TR = 2,600 ms; TE = 3.02 ms; TI = 900 ms, FOV = 256 mm^2^; FA = 8°). Echo planar imaging (EPI) was used to acquire resting-state data. For the 13-participant data set, two 6 min runs were acquired (33 interleaved axial slices; resolution = 3.4 × 3.4 × 3.6 mm^3^; TR = 2,000 ms; TE = 30 ms; FOV = 220 mm^2^; FA = 90°). For the 99 participants, only one resting state run was used per participant and there was minor variability across acquisition protocols with slice thickness, number of slices, TR, and scan duration ranging between 3.6 and 4 mm, 29 and 33 interleaved axial slices, 1,750 and 2,000 ms, and 6–10 min., respectively. Specifically: *n* = 56, slice thickness = 3.6 mm, 33 slices, TR = 2,000 ms, duration = 6.1 min; *n* = 36, slice thickness = 3.6 mm, 29 slices, TR = 1,850 ms, duration = 10.0 min; *n* = 7, slice thickness = 4.0 mm, 33 slices, TR = 2,000 ms, duration = 6.0 min. The resting state instructions were consistent throughout. Participants were instructed to keep their eyes open during the scan and direct their gaze on a white plus (+) sign centered on a black background. The verbal instructions before the scan were, “Remember to keep your eyes open and directed at the fixation symbol. Let your mind wander freely. If you notice yourself focusing on any particular train of thought, let your mind wander away from it.” Note that the Supplementary Material characterizes the quality of these two datasets. Supplementary Figures S1–S4 provide an overview of motion in terms of both displacement and displacement change for the *N* = 99 and *N* = 13 datasets.

Finally, while our findings were statistically robust, our 13-subject cohort is admittedly small. To evaluate the reproducibility of our findings we used 83 participants from an archival dataset (openneuro.org; Power et al., 2017) that included two resting-state runs per participant to replicate our cross-validated prediction accuracy estimates. These data were acquired at 3T (Siemens MAGNETOM Trio) with TR = 2,500 ms; TE = 27 ms; voxels = 4 x 4 x 4 mm; 32 interleaved slices. Run lengths varied from 130 to 133 volumes (mean: 132 volumes).

### Surface Masks

As shown in Figure 1 the training features are depth-limited signals. As such, this approach could be considered a surface-based analysis. The goal however is not to use sophisticated surface techniques such as those supported by FreeSurfer (Fischl, 2012) to reconstruct the topology of the cortical surface. Rather, the focus here for evaluating the potential future application to scalp-based measurements simply requires depth-limited masks of the brain's folded surface to simulate depth-attenuated measurements.

**Figure 2:**
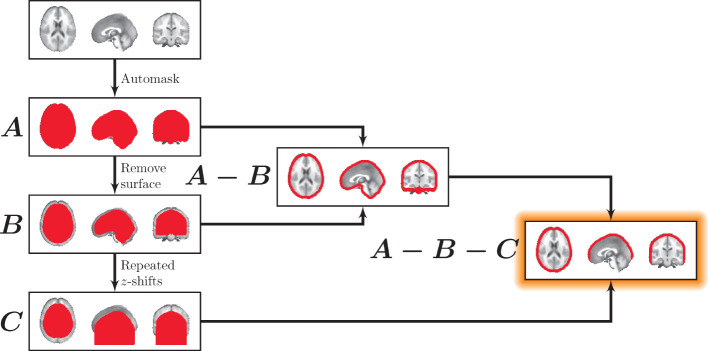
Masking the surface. First, a whole-brain mask was generated (*A*). All voxels within mask thickness *m* of the surface were eliminated to generate (*B*), containing the interior of the brain. A surface mask was then generated (*A*−*B*). To eliminate the inferior surface, (*B*) was repeatedly translated downward along the *z*-axis. After the first shift, each subsequent translational iteration was added to the previous one, creating an extended inferior volume shown in (*C*). Finally, *C* was used to exclude inferior regions in the final mask (*A*−*B*−*C*).

We used morphological image processing on binary whole-brain masks to obtain surface cortical masks. These surface masks excluded regions that would not be easily accessible to surface-based measurements (e.g., the inferior surface of the brain). The approach for deriving the surface masks is illustrated in Figure 2. Surface masks were developed from whole-brain binary masks that were zero-padded in the superior and inferior planes (Mask *A*). For mask thickness *m*, a “subsurface” mask was generated by zeroing each voxel within *m* millimeters from the surface boundary in any direction. That is, a new binary mask was produced that excluded voxels outside the brain as well as within *m* millimeters of the surface of the brain (see Mask *B*). Next a brain surface mask was generated by including all voxels within the whole-brain mask while excluding all voxels within the subsurface mask (Mask *A* − Mask *B*).

Finally, to exclude surface regions that are not easily accessible to surface-based methods (e.g., the cerebellum and the inferior surface of the brain), the subsurface mask was repeatedly translated in the inferior direction, and after the first shift, each subsequent translational iteration was added to the previous one, creating an extended inferior volume (Mask *C*). This effectively removed all voxels inferior to the cortex (Mask *A* − Mask *B* − Mask *C*). Note that for this study, we fixed the surface mask thickness, *m*, to 10 mm, which represents a conservative depth that is applicable to both MEG and fNIRS to explore the utility of surface-based tracking.

### Preprocessing

We used tools from AFNI (Cox, 1996) and FSL (Smith et al., 2004) to preprocess the imaging data. Specifically, FLIRT (Jenkinson et al., 2002) and FNIRT were used to register skull-stripped anatomical images to the MNI 152 space. Skull stripping was performed in AFNI and visually inspected for errors. We generated white matter and cerebrospinal fluid masks using a probability ≥ 0.99 threshold to both corresponding maps generated by the hidden Markov random field model methods implemented in FAST (Zhang et al., 2001). Resting-state data were slice-timing and motion corrected using the 3dTshift and 3dvolreg commands in AFNI, respectively. We used the mean image from each run to derive a linear transformation matrix to co-register each functional scan to that participant's corresponding anatomic image when using FLIRT. Next, nuisance variable regression was performed by regressing out white matter and cerebrospinal fluid time series, the six motion parameters calculated during motion correction, and a 4th order polynomial to account for baseline drifts (Friston et al., 1996; Lund et al., 2006). The functional-to-anatomical and anatomical-to-MNI152 transformations calculated for each dataset were concatenated to construct a functional-to-MNI152 transform. This transform was applied to the functional images, producing 4 mm isotropic data in MNI space. These images were then smoothed using a 6 mm^2^ full -width-at-half-maximum (FWHM) Gaussian kernel.

### Support Vector Regression

SVR models for the masked resting-state datasets were generated using 3dsvm (LaConte et al., 2005), a wrapper of SVM^light^ (Joachims, 1999) that is convenient for processing fMRI data. Our general approach was to normalize each subject's T1 anatomical volume to MNI space and extract a target time series from either a network or anatomical region. The target time series were generated from both resting state networks (RSNs) and atlas-defined regions, and the features consisted of either brain surface voxels or surface group map-based volumetric subdivisions. For the RSNs, we used the top 10 components from a 20-component ICA meta-analysis of resting state as well as task data by Smith et al. (2009) and obtained the target time series using spatial regression. The atlas-defined bilateral regions were extracted from the Eickhoff et al. (2005) macro-label atlas available in AFNI. We examined amygdala, anterior cingulate cortex (ACC), caudate nucleus, insula, posterior cingulate cortex (PCC), and putamen. The time series for the atlas-defined regions were calculated by extracting the ROI time series data from MNI space at 4 mm^3^ resolution and by averaging the time series data within each ROI. The time series targets served as labels for the surface features to generate SVR models using 3dsvm (Joachims, 1999; LaConte et al., 2005).

### Estimating Prediction Accuracy

Using the 13-participant dataset, each participant's two resting-state runs alternately served as training and testing data to produce a cross-validated (averaged) estimate of prediction accuracy. We defined prediction accuracy as the Pearson's correlation coefficient *r* between predicted and observed target time series. Appropriate SVR parameters were evaluated by Craddock et al. (2013); based on our previous work, *C* was set to 100 and ϵ was set to 0.1 with a linear kernel for surface-masked data.

To test the statistical significance of the prediction accuracies, we used a “wavestrapping” algorithm as outlined by previous groups (Breakspear et al., 2004; Bullmore et al., 2004). Specifically, a discrete wavelet decomposition of true target time series was used to generate 26,000 surrogate time series, that in turn were used to train SVR models and then predict the ‘true’ labels in the appropriate test data. The resulting set of SVR-based correlations provided a null distribution to non-parametrically estimate the *p*-value for each network and bilateral region. In other words, the “true training label”-based model was applied to the test data to produce a predicted time series of correlation accuracy *r*. Then the significance of *r* was estimated by training with the distribution of wavestrapped training labels to produce a distribution of correlations with the (unaltered) test data labels. Specifically the non-parametric *p*-value was approximated as the proportion of trials in which the true target's correlation exceeded the distribution of surrogate correlations.

The PyWavelets Python module (Wasilewski, 2006) was used to perform discrete wavelet decompositions on the target time series using the 4th-order Daubechies wavelet. Four levels were used, based on the equation


(1) $$\begin{array}{l} max\_level=\left\lfloor \log_{2}\frac{\text{signallength}}{\text{filterlength}-1}\right\rfloor , \end{array}$$

which ensures the signal is longer than the FIR filter length of the wavelet. Here, the signal contained 182 measurements and the decomposition filter length of the 4th-order Daubechies wavelet is 8. The detail coefficients, but not the approximation coefficients, at each level were randomly permuted. Then the resulting surrogate target time series was reconstructed with an inverse wavelet transform. As mentioned, the null distribution was constructed by repeating this process for 26,000 permutations for every network and region (an average of 1,000 permutations per run for every network and region) using GNU parallel (Tange, 2011). Finally, to evaluate the reproducibility of these results, we also estimated *r* values using archival data from 83 participants (openneuro.org; Power et al., 2017).

### Group Maps and Target-Specific Surface Subdivisions

In the 99-participant data set, a single resting-state run was used to generate surface SVR models. These results were subsequently used as first-level data to generate group maps for every RSN and bilateral region. The significant clusters within these group maps were then volumetrically subdivided to derive a sparse feature set for each target RSN and region. Each group map was organized into clusters of five or more nonzero voxels. Each voxel had to be within one grid cell of its nonzero neighbor to be included in the cluster. These clusters were recursively subdivided to ensure that their total volumes would be less than or equal to a fixed maximum volume, set to 40 voxels (2,560 mm^3^). To do this, the two points within a cluster with the greatest distance between them were found; these were used to derive a vector between them, ***v***, and a midpoint, ***x***. These were used to construct a plane normal to ***v*** and containing ***x***, bisecting the cluster: ***v***·***x*** = *b*. This procedure was used to generate two new clusters from each existing cluster. This subdivision procedure was repeated recursively through all clusters greater than 40 voxels in size. To use the subdivided surface as features, the voxel values within each subdivision were averaged. SVM^light^ was used directly and because these models had far fewer features, *C* and ϵ were set to the SVM^light^ defaults; *C* varied from model to model, whereas ϵ remained at 0.1.

### Summary of Methods

To summarize, we used 10 RSNs from Smith et al. (2009) as well as six bilateral anatomical regions. SVR, restricted to a 10-mm-thick surface of the brain, was used to model these 16 target signals. Data from 13 participants who completed two resting-state runs provided the ability to estimate prediction accuracy using split-half resampling (training on one run and testing on the other) (Strother et al., 2002). This was then replicated with additional 83-participant archival data set. To generate group maps of the surface, SVR training was performed on an additional 99 independent participants who completed only one resting-state run. For each target, the 99 SVR maps were combined using a one-sample *t*-test. Finally, to examine the impact of sparser and lower spatial resolution measures, these maps were volumetrically subdivided to create feature sets to also test with the 13- and 83-participant data sets.

## 3. Results

### Depth of Measurement

**Figure 3:**
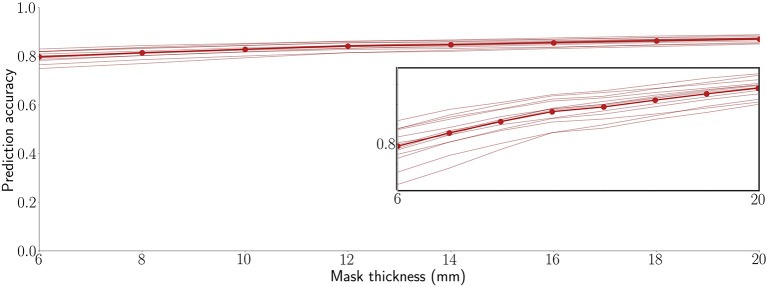
Decoding is highly accurate across a practical range of surface-limited recording depths. Each line is a participant's average across 16 target signals (10 RSNs and 6 anatomical regions). The bold line connected by points shows the average prediction accuracy across all 13 participants. The inset replicates the plot at a magnified vertical scale to emphasize that prediction accuracy increases monotonically with mask thickness and to highlight the observation that, even within the tight range of observed correlation values, participants are highly consistent in their performance ranking across the tested mask thicknesses.

This study used a mask thickness of 10 mm throughout. To characterize the impact of depth, however, Figure 3 shows the 13 participant SVR accuracies averaged across the 10 RSNs and 6 regions for mask thicknesses over a range of 5 to 20 mm. As shown, prediction accuracy is stable and high over this range, with no peak optimum or sign of asymptotic plateau. We observed that accuracy increases monotonically, with correlations increasing by approximately 0.005 per mm of mask thickness (*R*^2^ = 0.953). Interestingly, participants tend to rank by prediction accuracy. In other words, with only minor exceptions, the relative performance of each participant was consistent across all map thicknesses. Thus, some participants' brain activity was consistently easier to predict than others. This observation is examined further in our Supplemental Results (Supplementary Figure S5).

### Sparsity of Surface Features

**Figure 4:**
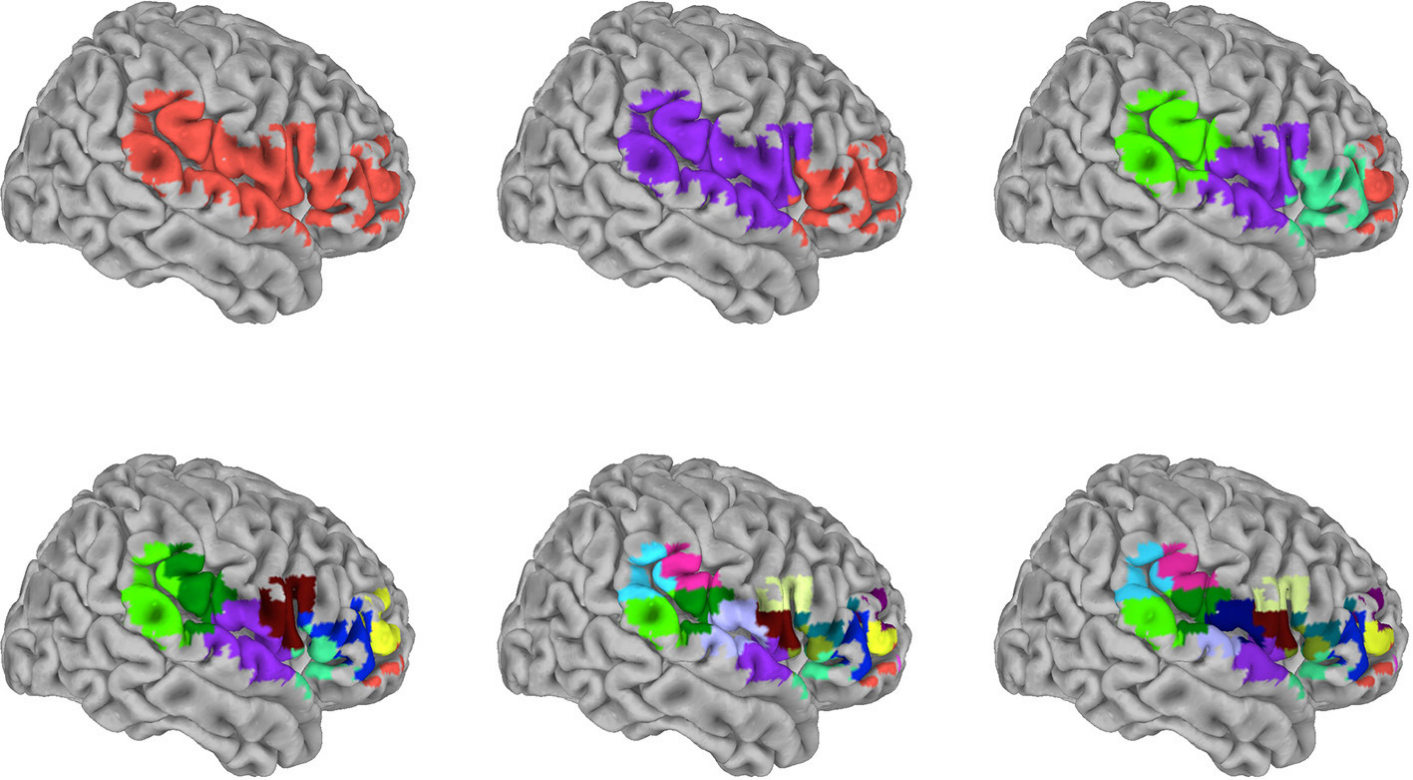
Graphical depiction of the iterative surface volumetric subdivision. Group maps were generated for each RSN and anatomical target signal using the 99-participant SVR surface models. Each significant cluster was then volumetrically subdivided to generate sparse feature sets for each target. This example shows the iterative steps used to subdivide cluster 1 in the insula target group map (see also Supplementary Table S14). The cluster spans frontal, postcentral, temporal, and parietal regions. This particular cluster was subdivided in 5 iterations, reducing 489 voxels to 17 subdivisions. Colors distinguish individual subdivisions. Subsequently, the signal average within each subdivision was used as a training/testing feature in the independent 13-participant data.

To examine sparser and lower spatial resolution measures, we volumetrically subdivided the SVR group maps for each of the 16 target signals. To do this, spatially distinct clusters were recursively subdivided to produce volumes of 2,560 mm^3^ (40 voxels) or less. The end result produced subdivided surface masks that were specific for each of the target signals. Figure 4 shows an example of the recursive volumetric subdivision for a surface cluster spanning right inferior frontal, superior temporal, and inferior parietal regions (from the insula target SVR group map). The Supplementary Tables S1–S16 include the volume and MNI coordinate for the centroid of each subdivision for all 16 target signals. For comparison, the full 10 mm surface models used approximately 8,000 voxels, and the subdivision produced an approximately 30–800 fold reduction of features (the number of subdivisions/reduced features were RSN1: 73, RSN2: 104, RSN3: 159, RSN4: 175, RSN5: 22, RSN6: 178, RSN7: 211, RSN8: 115, RSN9: 273, RSN10: 242, Amygdala: 10, ACC: 16, Caudate: 14, Insula: 50, PCC: 39, Putamen: 20). To test these subdivided features, training and test data from the 13-participant set were extracted by averaging within each member voxel of a given subdivision. This was then also examined in the replication data set (openneuro.org; Power et al., 2017).

### Decoding Performance, Surface Weight Vectors and Example Target Decoding

**Figure 5:**
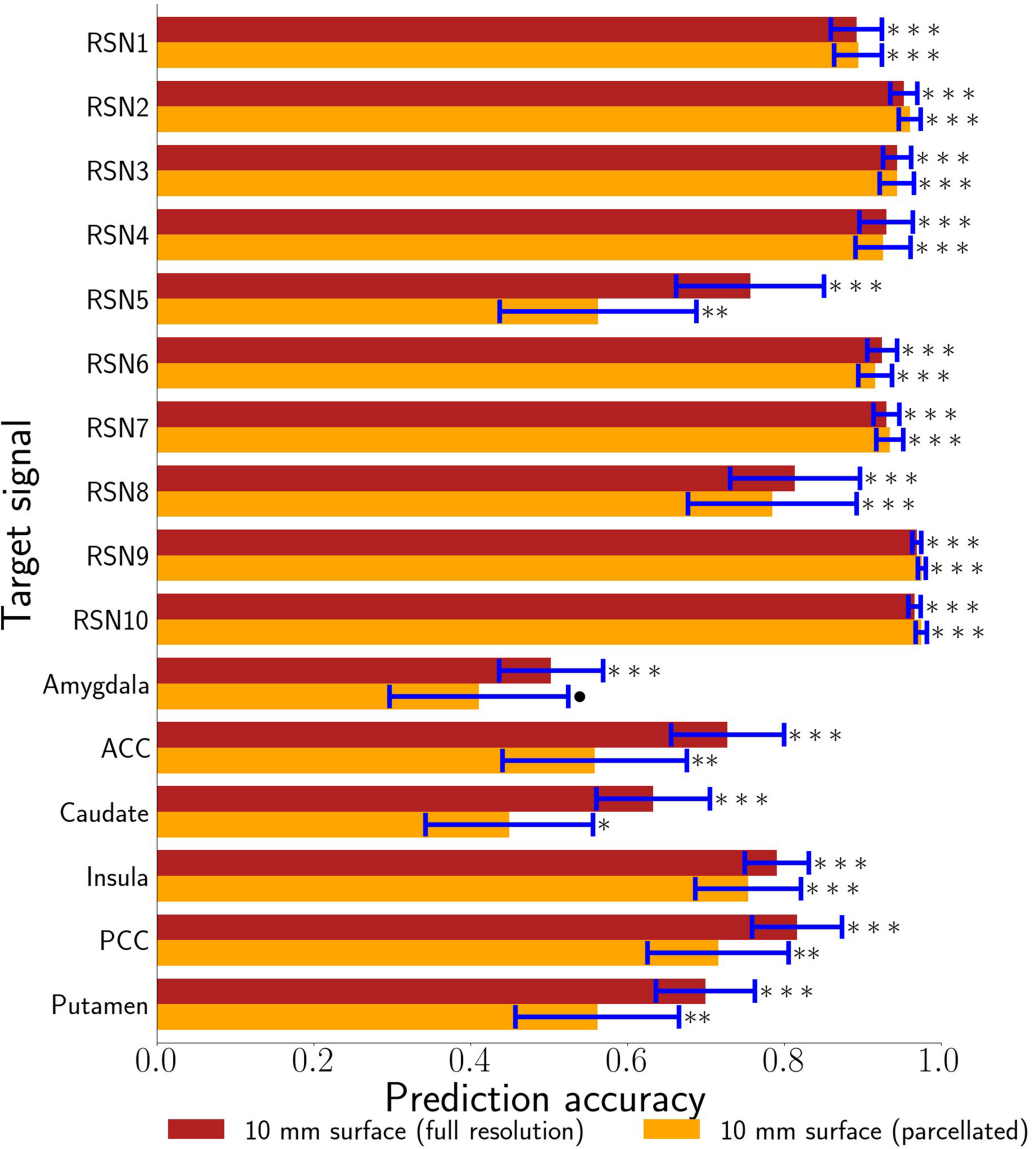
10 mm depth-limited prediction accuracies for all target signals demonstrates the general feasibility of tracking activity using the brain's surface. The full voxel resolution results are shown in maroon and the volumetrically subdivided results are shown in orange. Error bars are plus-or-minus one standard deviation. Non-parametric estimates of significance were generated from wavestrapped distributions and were corrected for multiple comparisons with respect to these 32 hypothesis tests. *** indicates corrected *p* < 0.001, ** indicates corrected *p* < 0.01, * indicates FDR-corrected *p* < 0.05, ∙ indicates corrected *p* < 0.06. ACC denotes anterior cingulate cortex. PCC denotes posterior cingulate cortex.

**Figure 6:**
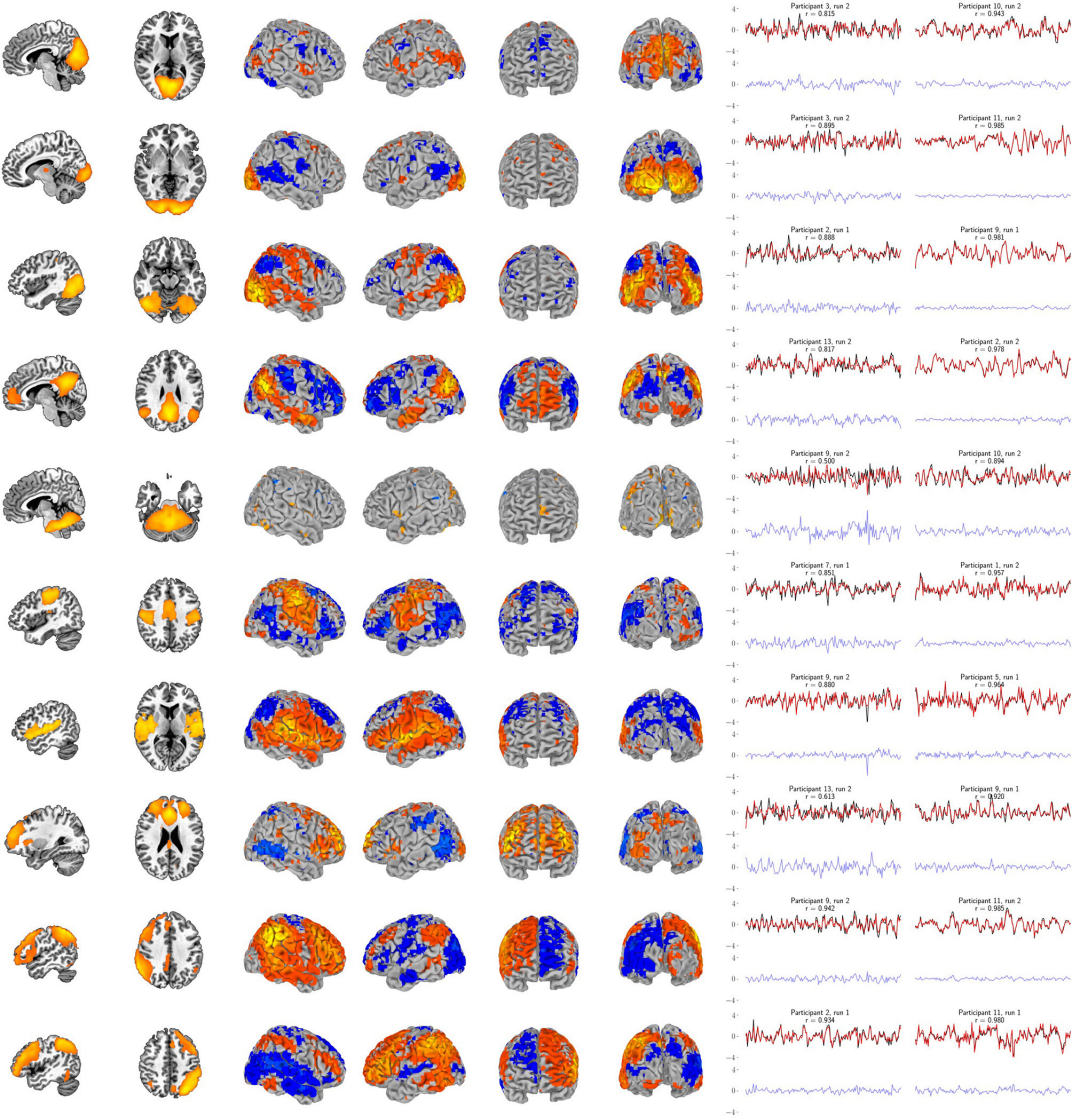
Support vector regression group maps for the ten resting-state networks from Smith et al. (2009). Left panel: The resting-state network templates (with RSN1 to RSN10 ordered from top to bottom, respectively). Center panel: Group maps from the 99-participant SVR models using the full-resolution (10 mm depth) mask for each participant (FDR-corrected *p* < 0.05). Group maps for all network targets are fully described in Supplementary Tables S1–S10. Right panel: The least and most accurate predictions for each target, respectively from the 13-participant data set. The actual target time series is black and the surface-limited prediction is red. Residual time series (target - predicted) are shown on the same scale below in blue. Annotations indicate the participant number, resting-state run number and correlation (*r*) between the target and predicted time series. For example, “Participant 1, run 2” indicates that a surface SVR model trained on that participant's run 1 data was used to decode her/his run 2 target activity (red time series). See Figure 7 for corresponding results for the six bilateral anatomical targets.

**Figure 7:**
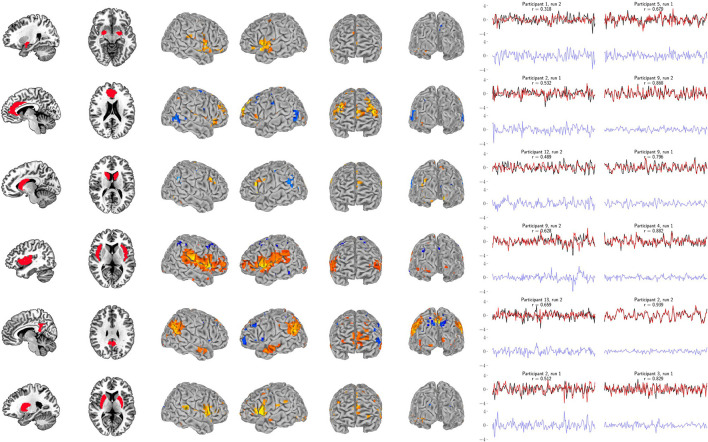
Support vector regression for the six bilateral anatomical targets. Left panel: Bilateral anatomical target regions. Ordered from top to bottom are amygdala, anterior cingulate, caudate, insula, posterior cingulate, and putamen. Center panel: Group maps from the 99-participant SVR models using the full-resolution (10 mm depth) mask for each participant (FDR-corrected *p* < 0.05). Group maps for all anatomical targets are fully described in Supplementary Tables S11–S16. Right panel: The least and most accurate predictions for each target, respectively from the 13-participant data set. The actual target time series is black and the surface-limited prediction is red. Residual time series (target-predicted) are shown on the same scale below in blue. Annotations indicate the participant number, resting-state run number and correlation (*r*) between the target and predicted time series. For example, “Participant 1, run 2” indicates that a surface SVR model trained on that participant's run 1 data was used to decode her/his run 2 target activity (red time series). See Figure 6 for corresponding RSN results.

Our primary question was, “Can we use the surface of the brain to track distributed networks and anatomical regions?” The prediction accuracies and wavestrap significance thresholds are shown in Figure 5, confirming that it is indeed possible to track interior brain regions and distributed RSN fluctuations using only surface measurements (see also Supplementary Figures S6, S7 for wavestrap details). For consistancy with our mapping results, Figure 5 uses MNI-normalized data. As Supplementary Figure S8 shows, however, the prediction accuracies are similar when calculated in in each subject's native (and unsmoothed) space. Moreover, these results were remarkably reproducible in the 83 participants (Supplementary Figure S9). Our secondary question was, “Can we map the surface connectivity that enables decoding?” Group maps of the 99 participants' surface weight vectors are shown for the RSN targets in Figure 6 and for the anatomical targets in Figure 7. These maps represent functional connectivity relationships between the surface and each predicted target signal. Specifically, these maps show consistencies in the SVR-derived multivariate combinations of surface time series that best reconstructed the interior brain signal. The maps in Figures 6, 7 are fully described in Supplementary Tables S1–S16 and available on neurovault.org (https://neurovault.org/collections/QAGSDTLT/).

In addition, these figures show the best- and worst-predicted run for the 13 participants, corresponding to the full resolution surface (maroon bars) in Figure 5. Finally, we examined the residuals of all predictions. Individual examples are include in Figures 6, 7. On average, the residuals appear to be normally distributed and absent of strong correlations between the residuals vs. predicted values. See Supplementary Figures S10, S11.

## 4. Discussion

The surface of the brain is comprised of the neocortex, an evolutionarily young, folded sheet of neurons that are the substrate for highly refined sensory, motor, and cognitive function. Its connectivity with itself and the rest of the brain is critical for higher order function and may be one of the keys to understanding neurodegeneration and psychiatric disorders. Importantly, since the cortex forms the exterior of the brain, all but its medial walls are accessible to a broad range of physiological measures. Consequently, however, the surface also physically obscures the deeper structures it surrounds to depth-limited measures. We hypothesized, though, that machine learning models of functional connectivity might enable the surface of the brain to be used as a steerable array, tuned to report activity of deeper regions and distributed networks. If this were true, then potentially even surface-limited measurements could be calibrated to report ongoing internal activity and to examine the multivariate information exchange between the cortex and the rest of the brain.

With this in mind, this study set out to evaluate whether supervised regression models of functional connectivity could harness multivariate signals from the surface of the brain to track underlying regions and distributed networks. Taken together, the results demonstrate that it is, indeed, possible to decode the brain's surface in such a manner. Thus, while physically surrounding the rest of the brain, the cortex nonetheless remains a window to the deeper activity it encases. These results reveal that a substantial amount of information is shared between the cortex and the rest of the brain through functional connectivity. The fidelity of the decoding depends upon several factors such as the depth of measurement, the specific target network or region, the trade-off between utilizing the entire surface vs. sparse, volumetrically-subdivided features, and the subject-specific variability observed in Figure 3. Note also that Figures 6, 7 indicate subject-specific characteristics for the best and worst predictions. Almost half of the worst performances (7 of 16) come from 2 of the 26 runs: participant 2's first run (ACC, putamen, RSN3, RSN10) and participant 9's s run (insula, RSN5/cerebellum, RSN9). Participant 9 is interesting, however, because while her/his second run represents three of the worst performances, her/his first run represents three of the best (caudate, RSN3, RSN8). Finally, although not shown here, we and others have previously noted that the amount of training data (in this case, the length of the fMRI runs) also plays an important role in determining the prediction accuracy of classifiers and the reliability of estimates (Kjems et al., 2002; LaConte et al., 2007; Pereira et al., 2009).

The RSN accuracies were generally higher than those for the bilateral anatomical regions included in this study. The results corroborate that these networks can, indeed, be treated as a unit and that the functional coherence that originally defined them (Smith et al., 2009) is replicated here in a new cohort and in a new analysis context. One important factor that also likely contributed to the strong performance of the RSNs is that their spatial extent partially comprises the surface, itself. Thus, there is a redundancy between the RSN voxels averaged to define the target signal and the surface features, vastly simplifying the training complexity. The exception is the cerebellar network (RSN5), which is also notable in that it demonstrated the largest performance drop when going from the full surface model to the volumetrically subdivided one. This suggests that the 99-subject group model does not generalize as well for RSN5 and that more extensive surface coverage aids in predicting cerebellum when it is defined as a cohesive network. Future studies are needed to further characterize functional connectivity of the cortex with anatomical and functional sub-regions of the cerebellum. Similar considerations are warranted for the six bi-lateral anatomical regions. It is possible that their accuracy might be improved by modeling smaller, unilateral subregions. From a signal-to-noise point of view, it might be ideal to define target signals by averaging across regions or networks that have optimized the statistical trade-offs between maximizing spatial extent and maximizing signal homogeneity (Shirer et al., 2012). On the other hand, mixtures of signal sources may or may not degrade accuracy, depending upon how well this mixture can be modeled from the surface features. Thus from a more nuanced view, both the bi-lateral anatomical regions and the RSNs used in this study can be considered as networks. Further, it is important to recall that fMRI voxels are several orders of magnitude larger than their underlying cellular processes and are thus fundamentally mixtures of signal sources. Related to these issues, further improvements could possibly come from adaptations of hyperalignment (Haxby et al., 2011; Feilong et al., 2021), which could be used to account for individual variations in coarse-grained patterns and to maximize the similarity within ROIs and networks as well as with the brain's surface. Overall, we expect that tracking both smaller subdivisions as well as extensive distributed networks will be important in future research. Additional studies will be needed to comprehensively test the factors that determine how well any given target signal can be tracked from the surface. To summarize, all regions and networks are a mixture of several sources and prediction accuracy is a metric to evaluate how functionally connected to the cortex the chosen target signal is. At present, the broad success of all sixteen target signals and thus the conclusion that these are all simultaneously represented at the level of surface cortex is fascinating.

fMRI proved to be ideal for demonstrating the feasibility of surface-based tracking. Because of its high resolution, it enabled examination of the extremes of surface density measurements to evaluate the theoretical impact of high and low numbers of training features. And in the majority of cases, high accuracy was maintained with a much smaller feature space. In addition, validation is possible because the fMRI data are whole-brain. The two runs in the 13-participant data set and in the 83-participant replication set allowed us to estimate our primary measure, surface-based decoding accuracy, in a cross-validation framework (alternating the role of training and testing runs and averaging the two prediction accuracy estimates). For today's technology, the high accuracy reported here likely serves as an optimistic upper bound on what could be accomplished in an actual multimodal setting. As pointed out by the reviewers of this work, spatial correlations, head motion, global signal, and physiology are all sources of noise that may need to be further investigated. Moreover, the relative impact of each of these will almost certainly vary from modality to modality, and thus may need different strategies. One interesting possibility, however, is that variations on our approach may lead to new ways to incorporate such nuisance signals directly into the multi-modal modeling approach. As shown in Supplementary Figures S10–S11, the residuals from our surface models appear to be largely white noise—notably unlike any of the confounding potential noise sources that we have listed. Thus, in our case, the preprocessing that we applied combined with the supervised learning process seemed to effectively regress out these confounding signals. Another point that should be elaborated upon is that the goal of our study deviates from what some would consider usual notions of data independence. For example, many of the RSNs extend to the brain's surface. Rather than excluding surface data features, we actually want them to be incorporated into the training stage. As shown in Figure 6 these surface portions of the networks are highly likely to be important features in the model. But what Figure 6 also shows is that the models do not exclusively rely on such regions. Indeed if they did, that would be a sign that just that region and not its *network* was being disproportionately modeled. As Figure 6 shows, this is not what is observed. Even RSNs 1–3, which are close to the occipital pole, converge to highly distributed surface patterns. Finally, along these lines, partial volume effects for fMRI data could partially serve as an aid or confound for tracking regions with surface measures. Analogous to partial volume concerns, each surface modality has its own individual depth sensitivity profile. Thus, while this study demonstrates the feasibility of using surface models to track deeper regions and networks, future work will be needed to address specific concerns for adopting this for actual surface recordings, and validation of such future work will likely require simultaneous multimodal measurements. Finally, while our stated goal was to demonstrate a simple approach to surface modeling and specifically to preserve the folded surface, it is possible that future improvements could come from more sophisticated techniques. As the reviewers have pointed out, with volume-based registration brain voxels around the cortical surface can suffer from severe misalignment issues. While a careful comparison would constitute a study of its own, it is possible that surface-based registration may be a necessary future step for future multi-modal implementations. At this point, however, our intuition is that challenges with surface probe/sensor placement will constitute a larger source of future variance.

In a complementary way, the 99-participant data set provided ample training data to examine commonalities of the SVR models across individuals. As the maps show (center panels in Figures 6, 7), this analysis approach has a secondary benefit as a tool for studying functional connectivity with the cortex. And, since the volumetrically-subdivided masks that these group maps generated were independent from the 13- and 83-participant sets, we also had the opportunity to go beyond mapping and validate the predictive potential of these functional connectivity findings. On the whole, the group maps representing the surface models of the 6 anatomical regions and the 10 RSNs are highly informative. Since the cortex's functional connectivity is thought to be one of the enabling properties of higher order function, this methodological approach could provide a new avenue for precisely exploring specific, restricted connectivity questions.

For example, the amygdala's functional roles and its connectivity with the brain's surface are continually being refined. The results in Figure 7 (see also Supplementary Table S11) overlap with frontal and temporal regions reported by Bickart et al. (2012, 2014), who explore this region's role as a critical hub of the social brain. This is just one recent case illustrating how our understanding of the function of the amygdala and its connectivity with the brain's surface continues to evolve. The first comprehensive description of the cortical projections of the amygdala in primates was reported by Amaral and Price (1984), who also described the early history uncovering its cortical projections. As they note, amygdalo-cortical connections were neither widely known nor appreciated until the early 1960s. As the complexity of the amygdala's connectivity was gradually discovered, so too was the recognition that its functional role was also likely to be highly complex. Thus, while our driving question was “Can limited surface models be used to track networks and deeper brain regions?,” our approach also doubles as a mapping tool to explore functional connectivity with the brain's surface.

Our analysis treats the cortex as a tunable receiver array, but this conceptual framework should not be taken literally, and it is important to note that the direction of information exchange between the surface and other brain regions cannot be resolved with our approach. It is likely that these models reflect both the cortex's signals to and from other regions. The ability to examine functional connectivity and to use this property to track time series derives directly from previous work on multivariate functional integration (Friston et al., 1993; Friston, 1994; Chu et al., 2011; Craddock et al., 2013). This approach has been applied to examine functional hierarchies using both connectivity maps and prediction accuracy (Craddock et al., 2013) through the “non-parametric prediction accuracy, influence, and reproducibility resampling” (NPAIRS) framework (Strother et al., 2002; LaConte et al., 2003). It is important to note that our previous results (Craddock et al., 2013) are not directly comparable to the those reported here. In Craddock et al. (2013) we used parcellated features throughout the entire brain, including subcortical areas, while this study used features restricted to the most superficial surface of the brain. So, for example, our full-resolution amygdala results have an average prediction accuracy of *r* = 0.5 and our sparse surface features give *r* = 0.41, while the subjects in Craddock et al. (2013) average about *r* = 0.8. This reflects expected decreases in accuracy because much of the amygdala's functional connectivity is unavailable to the surface-restricted models explored here.

Additional studies of functional integration include Hay et al. (2017) who demonstrated that minimal sets of features could be derived with recursive feature elimination and used to quantify regional degree of functional integration. Further, multivariate functional integration model weights have been demonstrated as a means of connectotyping (or functionally fingerprinting) individuals since they produce a personalized model-based connectivity matrix (Miranda-Dominguez et al., 2014). In this current study we have demonstrated the utility of restricting the functional integration investigation through the use of surface training features. Several additional variations of this approach are possible to study the functional integration between combinations of functional or anatomical nodes and networks which would complement graph theoretic (Bullmore and Sporns, 2009) and dynamic causal modeling (Friston et al., 2003) studies. Though not explored here, the utility of functional integration may be enhanced by comparing and contrasting different loss functions provided by alternative regression techniques such as LASSO (Tibshirani, 1996) and elastic nets (Zou and Hastie, 2005).

Restricting the training features to the surface of the brain enabled us to computationally assess the feasibility of multimodal enhancements to depth-limited measurements. Three major factors interplay when considering the possibility of using surface recordings to reconstruct deeper activity. The first (which was the primary focus of this work) concerns algorithmic and data properties, such as the density of measurements and their distributed coverage. A common goal in machine learning is to capture as much information as possible with the fewest possible data features. For practical reasons, it is desirable to assess the impact of sparse recordings as well as the impact on depth of sensitivity. fMRI is well-suited to study these issues since it provides whole brain coverage at a relatively high spatial resolution, surpassing what is currently achievable in terms of both density and depth of surface recordings. The second factor is the structure of the cortex, itself. Although its flattened dimension is approximately 2.5 mm thick on average, ranging from 1 to 4.5 mm (Fischl and Dale, 2000), its folded sulcal depths in humans have been reported to range from 1 to 3 cm (Ribas, 2010). Thus, the relative distance and orientation of gray matter varies dramatically as a function of position along the surface of the brain. Looking forward to future studies, the third factor is that each surface measurement has unique physical limitations. For example, MEG records magnetic fields outside the head that are generated by neural currents. Its sensitivity depends on both the depth and orientation of the generating current sources (Goldenholz et al., 2009). In contrast, functional near-infrared spectroscopy (fNIRS) uses two near-infrared wavelengths to assess changes in blood oxygenation arising from neural metabolic demands. Estimates of fNIRS penetration depth varies in the literature, ranging from 12 to 21 mm into the brain (Okamoto et al., 2004; Schroeter et al., 2006; Lu et al., 2010).

Moving beyond the feasibility of surface-limited tracking demonstrated here, implementation could take several forms. The basic notion is that a modality such as fMRI (that can measure both target and surface training signals) be used to calibrate a surface-limited modality such as fNIRS, MEG, or EEG. Such a process would enable those modalities to track activity that far surpasses their inherent physical capabilities. Early empirical work suggests that this is possible in EEG, in which amygdala activity can be tracked using an EEG finger-print (Meir-Hasson et al., 2014) and in fNIRS, where similar analyses confirmed the ability to track deep brain regions in task-based fMRI (Liu et al., 2015). Thus, there is early positive evidence suggesting that it is possible to dramatically enhance surface limited methods well beyond their intrinsic physical capabilities. In the meantime, we have additionally shown that the brain has a rich surface connectivity that can be mapped and has simultaneous predictive potential for decoding a range of distributed networks as well as interior, localized regions.

## Data Availability Statement

The datasets presented in this study can be found in online repositories. The names of the repository/repositories and accession number(s) can be found below: https://neurovault.org/collections/QAGSDTLT/.

## Ethics Statement

The studies involving human participants were reviewed and approved by Virginia Tech IRB. The patients/participants provided their written informed consent to participate in this study.

## Author Contributions

MT: investigation, methodology, software, and writing. JL: investigation, methodology, software, data curation, and writing. SL: investigation, methodology, supervision, and writing. All authors contributed to the article and approved the submitted version.

## Funding

The authors would like to thank the Virginia Tech Open Access Subvention Fund for supporting the publication costs of this article.

## Conflict of Interest

The authors declare that the research was conducted in the absence of any commercial or financial relationships that could be construed as a potential conflict of interest.

## Publisher's Note

All claims expressed in this article are solely those of the authors and do not necessarily represent those of their affiliated organizations, or those of the publisher, the editors and the reviewers. Any product that may be evaluated in this article, or claim that may be made by its manufacturer, is not guaranteed or endorsed by the publisher.
