# Supplementary material for: Decoding the Brain's Surface to Track Deeper Activity
Source: Front Neuroimaging. 2022 Mar 17;1:815778. doi: 10.3389/fnimg.2022.815778 (PMC10406232; doi:10.3389/fnimg.2022.815778)
Supplement: Supplementary file 1 [file Data_Sheet_1.PDF]

Supplementary Material

1 SUPPLEMENTARY DATA

1.1 Preprocessing and data quality

Figures S1–S4 provide an overview of motion in terms of both displacement and displacement change for the $N = 99$ and $N = 13$ datasets.

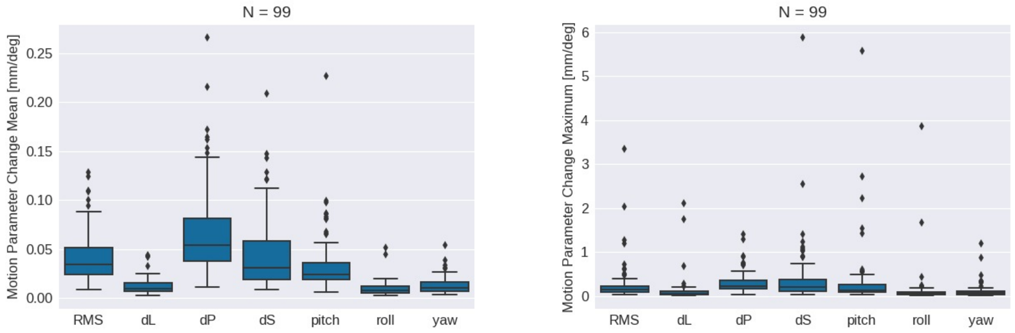

Figure S1. Mean and maximum displacement change ($N = 99$).

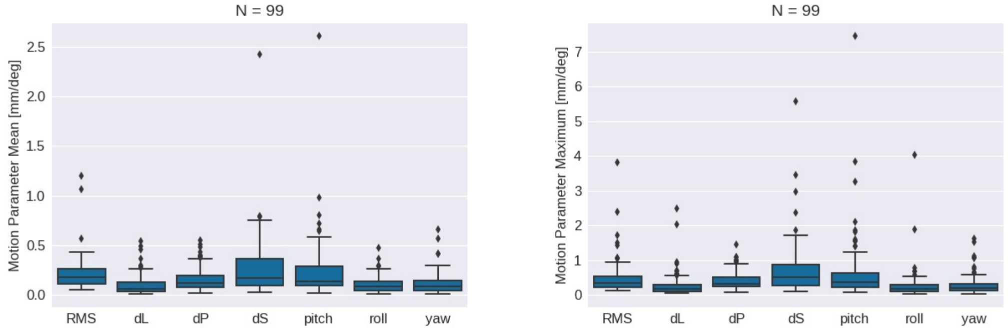

Figure S2. Mean and maximum displacement ($N = 99$).

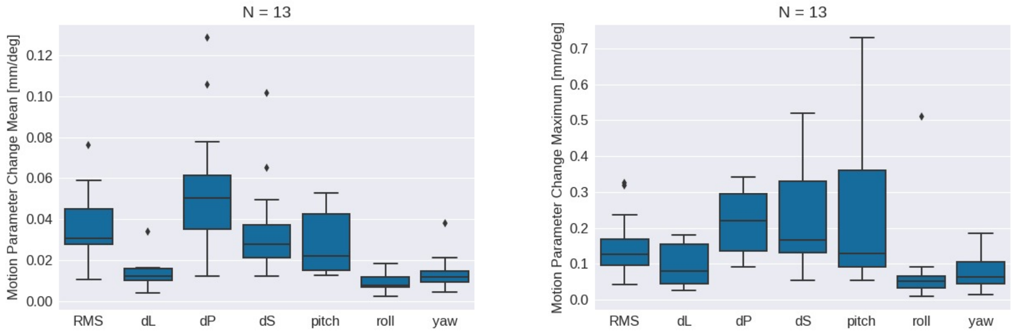

Figure S3. Mean and maximum displacement change ($N = 13$).

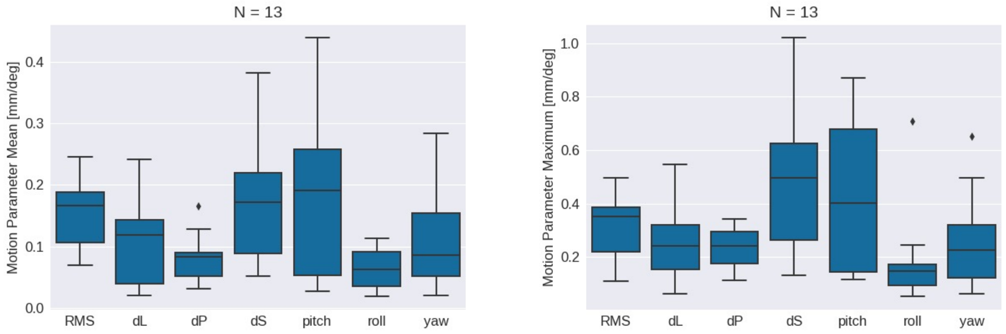

Figure S4. Mean and maximum displacement ($N = 13$).

1.2 Role of participant variability in prediction accuracy

Figure S5 shows the distribution of accuracy across the 13 participants, illustrating that some participants' brain activity was easier to predict than others.

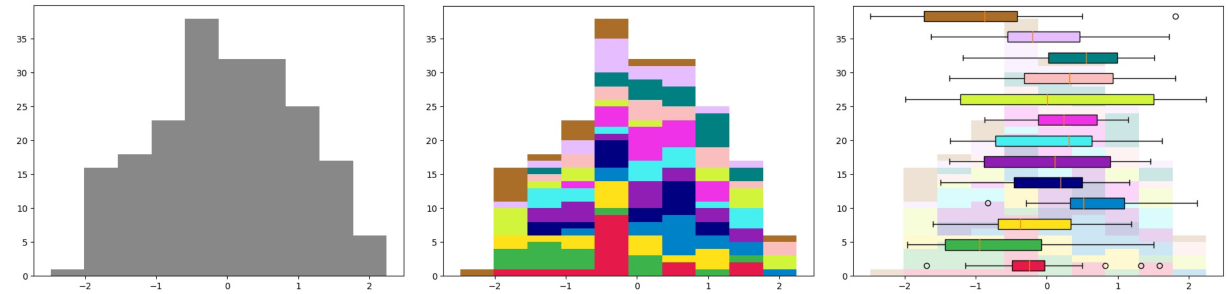

Figure S5. Distribution of prediction accuracy across the 13 participants; each accuracy value is z-scored with the mean and standard deviation for that RSN or brain region to isolate inter-participant differences. (Note that z-score normalization 'equalizes' the fact that the RSN accuracies were high compared to the anatomical regions.) In the left panel, the overall distribution is shown; in the center, each participant is shown within this distribution with a unique color. The revealed inter-participant differences are highlighted in boxplots overlaid on the histogram (right panel).

1.3 Wavestrap-generated null distributions

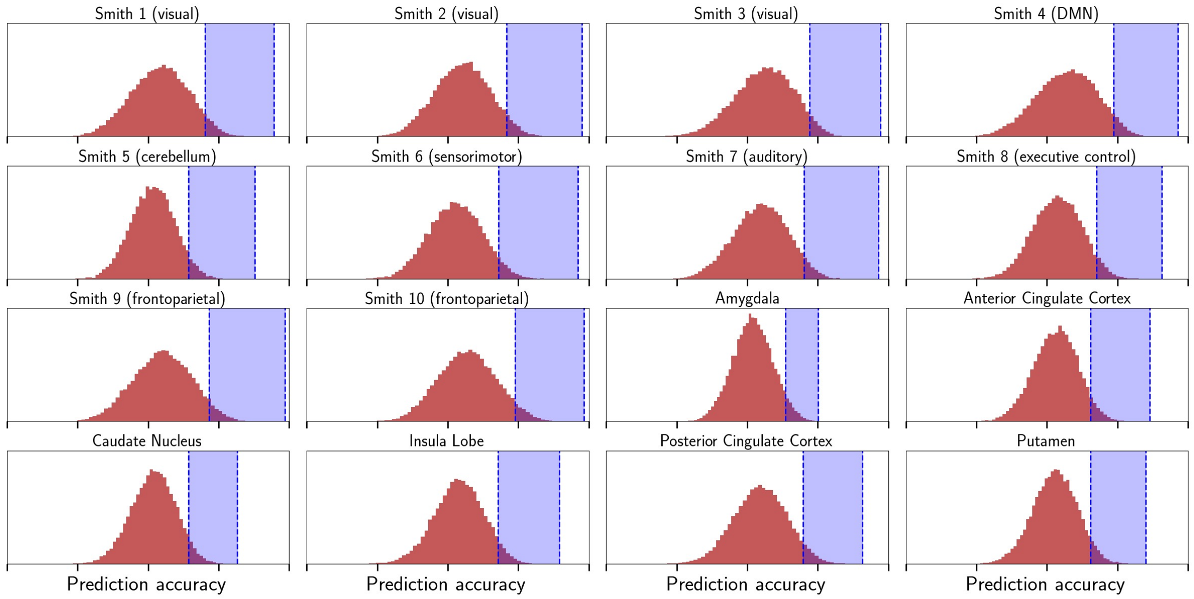

Figure S6. Full surface. Histograms of the null distributions of full-resolution 10 mm surface models, plotted on identical x and y-axes for comparison. The margin between the minimum prediction accuracy to pass uncorrected statistical significance (the 95th percentile of the null distribution, corresponding to uncorrected $\alpha = 0.05$) and the true prediction accuracy r is shaded. Wider blue margins correspond to greater statistical significance.

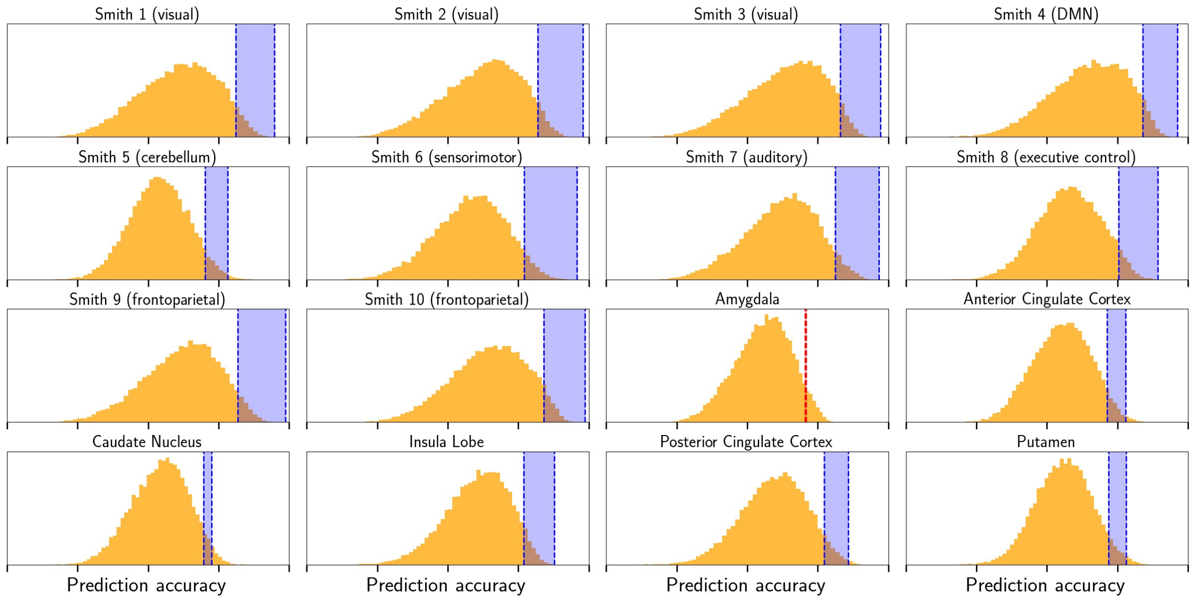

Figure S7. Sparce surface features. Histograms of the null distributions of sparse surface feature models, plotted on identical x and y-axes for comparison. The margin between the minimum prediction accuracy to pass uncorrected statistical significance (the 95th percentile of the null distribution, corresponding to uncorrected $\alpha = 0.05$) and the true prediction accuracy r is shaded; blue indicates statistical significance, and red indicates the lack thereof. Wider blue margins correspond to greater statistical significance.

1.4 Normalization and smoothing

Figure S8 the prediction accuracies cross-validated are neither strongly nor systematically affected by normalization and smoothing.

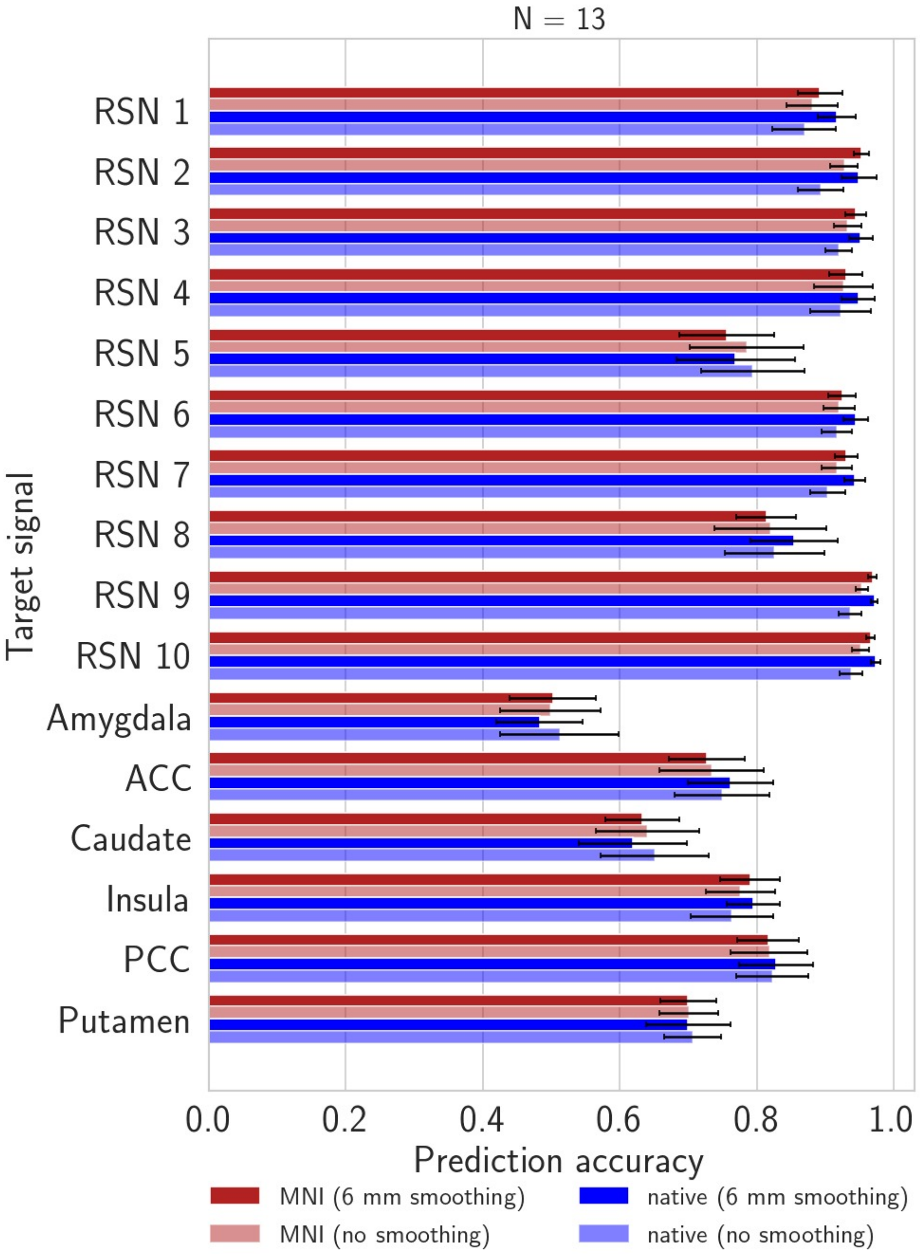

Figure S8. Normalization and smoothing. As shown the results from the 13-participants (Fig. 5 results shown again here for convenience) were neither significantly nor systematically different when calculted in unsmoothed, native space data (as well as in the other two combinations of these factors).

1.5 Replicating prediction accuracy

Figure S9 the cross-validated replication results for both the 13- and 83- participant data sets.

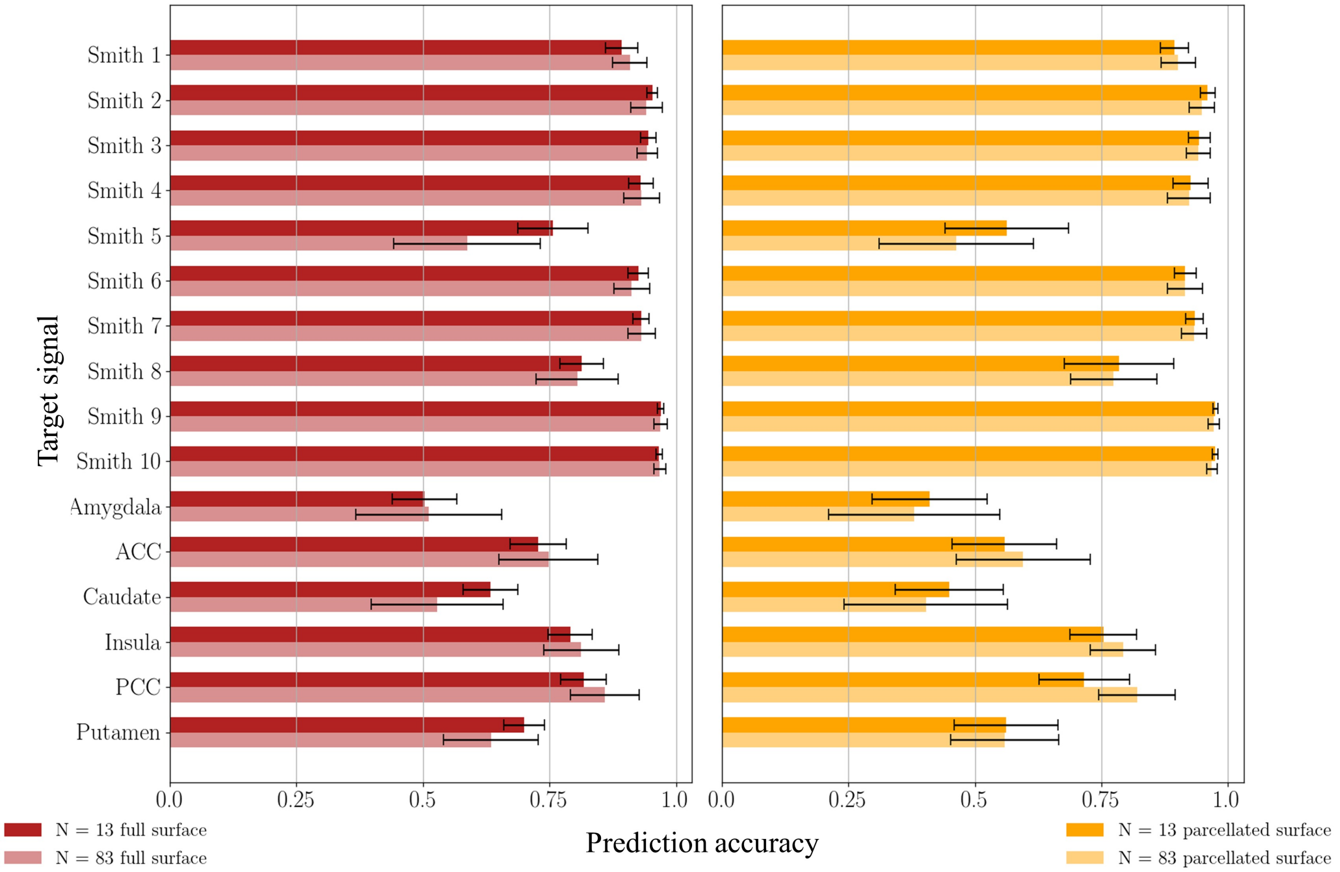

Figure S9. Replication of the primary result. As shown the results from the 13-participants (Fig. 5 results shown again here for convenience) were replicable with the 83-participant data set (openneuro.org; Power et al. (2017)). Shown are the 10 mm depth-limited prediction accuracies for all target signals. (Wavestrap estimates of statistical significance were not performed). Error bars are plus-or-minus one standard deviation.

1.6 Inspecting residuals

The residuals for the prediction accuracies in Fig. 5 for the full resolution surfact data are shown in Figs. S10 and S11.

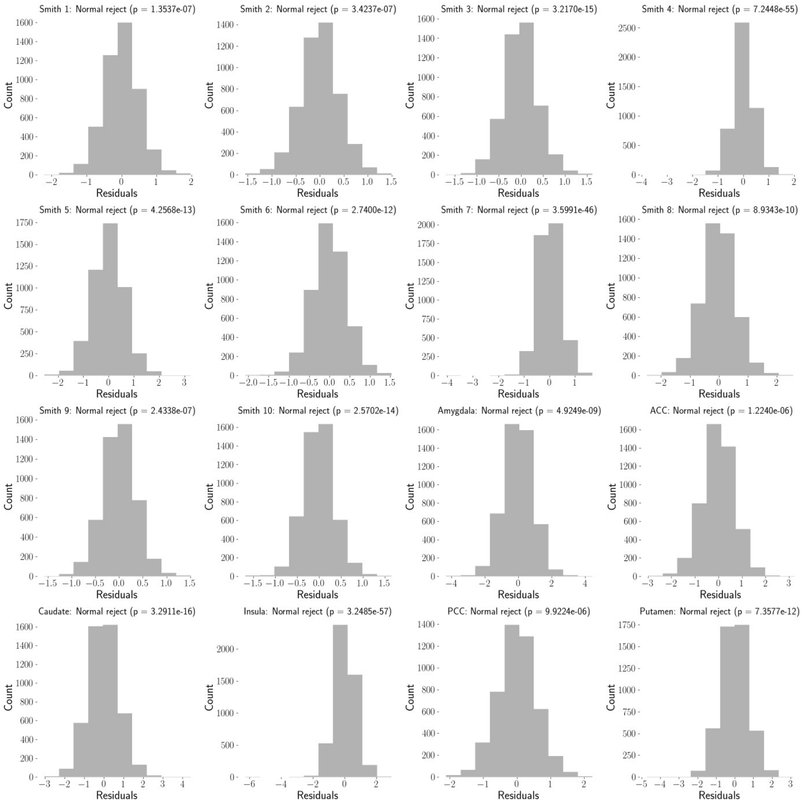

Figure S10. Histograms of residuals. The histograms were generated by subtracting the ROI/RSN label from the SVR predictions for both runs in the 13-participant data set. All histograms shown appear to be normally distributed, with each title including the (highly statistically significant) p-value assosciated with the normality test implemented in scipy D'Agostino (1971); D'Agostino and Pearson (1973).

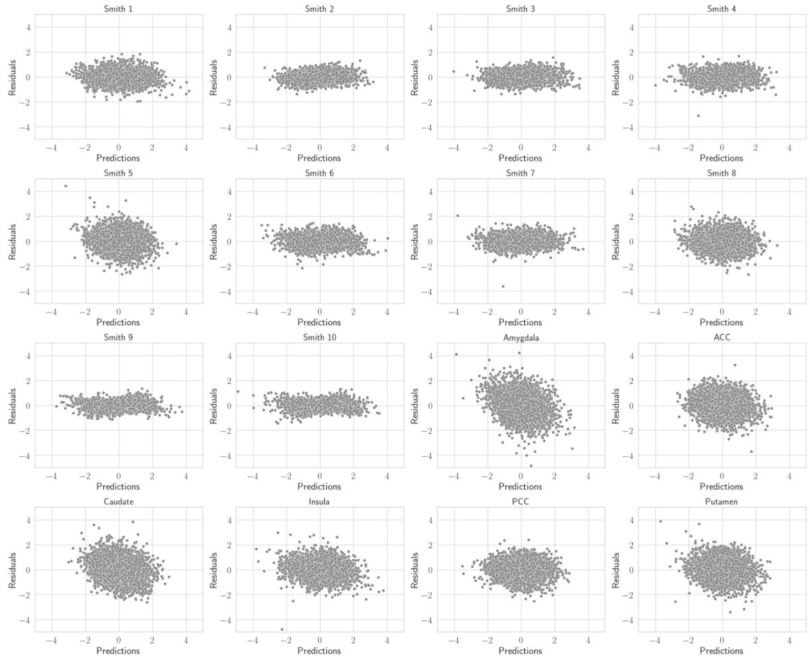

Figure S11. Scatterplots of residuals. Each plot shows the residual vs. the predicted value for each timpoint of both runs for the 13-participant data set. The spherical and elongated oval shapes that are present in all plots are consistent with the normal distributions seen in Fig. S10. Some of the poorer predicting RSNs and ROIs had weak linear deviations in their spherical clouds. For example, RSN5 had a correlation value of 0.08, amygdala had a correlation of 0.3 and caudate had a correlation of 0.2.

2 SUPPLEMENTARY TABLES

The 99-participant SVR group maps produced clusters indicated by integers in the tables below (e.g., “1. Bilateral Occipital” and “2. Right Occipital/Temporal”). For these clusters, the coordinates indicate their statistical peaks. Each of these clusters were also recursively subdivided volumetrically to produce subdivisions of 2560 mm^3 (40 voxels) or less. These subdivisions are indicated by decimal numbers (e.g., “2.1 Right Fusiform Gyrus”). The coordinates for these subdivisions represent their geometric centroids. Coordinates are given in MNI space and RAI/DICOM orientation, and t-values are peaks within the region.

Note also that the maps corresponding to these tables have been uploaded to neurovault.org (`https://neurovault.org/collections/QAGSDTLT/`).

Table S1: Target network: Smith et al. 1 (visual).

	Cluster Subdivisions	BA	Voxels	Location			Peak
				x	y	z	t
**1**	**Bilateral Occipital Cluster**	19	764	1	88	17	15.05
1.1	Left Cuneus	17	33	0	101	-11	
1.2	...	19	31	4	89	31	
1.3	...	19	28	17	94	23	
1.4	...	19	28	5	94	20	
1.5	...	18	19	8	103	1	
1.6	...	17	19	9	104	-8	
1.7	...	19	15	23	89	27	
1.8	Left Middle Occipital Gyrus	19	28	27	93	13	
1.9	...	18	27	8	99	10	
1.10	...	19	24	45	85	1	
1.11	...	19	21	42	83	12	
1.12	Left Middle Temporal Gyrus	39	35	51	71	11	
1.13	Left Precuneus	19	31	13	84	38	
1.14	...	7	23	1	82	42	
1.15	Left Superior Temporal Gyrus	22	29	60	55	15	
1.16	Right Cuneus	19	36	-23	86	30	
1.17	...	17	29	-13	101	-5	
1.18	...	18	28	-12	95	19	
1.19	...	19	27	-21	92	23	
1.20	...	18	23	-2	98	10	
1.21	...	19	20	-4	85	36	
1.22	...	18	18	-18	99	4	
1.23	...	19	16	-10	92	29	
1.24	...	19	15	-3	89	25	
1.25	Right Middle Occipital Gyrus	19	35	-30	88	13	
1.26	...	19	28	-48	75	5	
1.27	...	18	26	-11	98	10	
1.28	Right Middle Temporal Gyrus	39	17	-44	78	15	
1.29	Right Precuneus	19	28	-18	82	39	
1.30	...	7	27	-9	79	43	
**2**	**Right Occipital/Temporal Cluster**	37	65	-57	63	-16	-7.31
2.1	Right Fusiform Gyrus	19	20	-54	71	-18	
2.2	Right Inferior Temporal Gyrus	20	24	-62	54	-21	
2.3	Right Middle Occipital Gyrus	19	21	-57	67	-11	
**3**	**Right Frontal Cluster**	44	52	-59	-4	25	-5.77
3.1	Right Inferior Frontal Gyrus	45	23	-56	-19	22	
3.2	Right Precentral Gyrus	4	29	-62	7	27	
**4**	**Left Occipital Cluster**	18	47	32	98	-11	-4.84

Target network: Smith et al. 1 (visual - continued).

	Cluster Subdivisions	BA	Voxels	Location			Peak
				x	y	z	t
4.1	Left Inferior Occipital Gyrus	18	33	33	97	-16	
4.2	Left Middle Occipital Gyrus	18	14	32	101	0	
**5**	**Left Frontal Cluster**	10	45	0	-59	28	-5.07
5.1	Left Superior Frontal Gyrus	9	28	1	-57	33	
5.2	Right Medial Frontal Gyrus	10	17	-4	-65	20	
**6**	**Left Precentral Gyrus**	44	44	58	14	35	-4.79
6.1	...	6	32	57	-2	29	
6.2	...	4	12	58	14	35	
**7**	**Right Superior Temporal Gyrus**	21	43	-63	-9	-2	4.72
7.1	...	22	23	-64	7	0	
7.2	...	22	20	-63	-9	-2	
**8**	Left Cuneus	19	20	32	89	35	-4.78
**9**	Left Inferior Parietal Lobule	40	10	46	56	53	3.87
**10**	...	40	7	65	37	29	3.30
**11**	Left Inferior Temporal Gyrus	20	7	65	11	-27	-3.54
**12**	Left Middle Frontal Gyrus	6	8	29	-1	63	3.30
**13**	...	9	6	52	-19	39	4.55
**14**	...	9	6	39	-36	38	3.71
**15**	...	10	5	40	-56	16	3.38
**16**	Left Middle Occipital Gyrus	19	17	50	74	-19	-5.10
**17**	Left Precentral Gyrus	4	25	17	34	76	4.63
**18**	...	4	10	50	9	52	4.51
**19**	Left Superior Frontal Gyrus	9	8	11	-49	42	-4.15
**20**	...	6	5	10	-4	71	3.99
**21**	Left Superior Temporal Gyrus	22	33	62	-5	1	4.84
**22**	...	22	29	65	23	11	4.78
**23**	Left Tuber		6	45	80	-43	4.23
**24**	Right Angular Gyrus	39	37	-49	75	32	-5.72
**25**	Right Inferior Frontal Gyrus	46	14	-52	-39	7	-4.26
**26**	Right Inferior Parietal Lobule	40	12	-50	38	54	-3.59
**27**	Right Inferior Temporal Gyrus	20	10	-66	8	-30	-3.79
**28**	Right Lingual Gyrus	18	36	-34	95	-15	-4.25
**29**	Right Medial Frontal Gyrus	6	15	-13	-2	68	4.20
**30**	Right Middle Frontal Gyrus	46	12	-44	-47	30	4.64
**31**	Right Middle Occipital Gyrus	18	8	-38	94	13	-4.08
**32**	Right Postcentral Gyrus	5	6	-9	46	69	4.30
**33**	Right Precentral Gyrus	6	22	-50	4	53	4.91
**34**	Right Superior Parietal Lobule	7	6	-29	67	52	-3.67
**35**	Right Superior Temporal Gyrus	40	6	-62	47	19	3.35
**36**	Right Tuber		19	-44	82	-42	4.67

Target network: Smith et al. 1 (visual - continued).

	Cluster Subdivisions	BA	Voxels	Location			Peak
				x	y	z	t
**37**	…		5	-52	72	-34	3.94

Table S2: Target network: Smith et al. 2 (visual).

	Cluster Subdivisions	BA	Voxels	Location			Peak
				x	y	z	t
**1**	**Bilateral Occipital Cluster**	18	1286	0	95	-2	21.17
1.1	Left Cuneus	18	38	10	100	17	
1.2	...	19	36	20	95	25	
1.3	...	17	36	5	103	-7	
1.4	...	18	30	13	109	-1	
1.5	...	18	29	19	104	9	
1.6	...	18	27	23	103	-4	
1.7	...	18	20	22	108	-9	
1.8	...	18	18	9	102	7	
1.9	Left Declive	18	38	43	85	-25	
1.10	Left Fusiform Gyrus	18	25	31	97	-23	
1.11	Left Inferior Occipital Gyrus	17	31	28	102	-15	
1.12	...	18	25	40	90	-12	
1.13	...	18	17	39	96	-9	
1.14	Left Lingual Gyrus	18	35	9	107	-14	
1.15	...	17	24	14	104	-21	
1.16	Left Middle Occipital Gyrus	19	36	26	99	14	
1.17	...	18	30	50	78	-4	
1.18	...	19	29	50	76	-14	
1.19	...	18	29	39	89	6	
1.20	...	18	28	32	97	6	
1.21	...	18	25	34	99	-2	
1.22	...	19	19	46	87	-3	
1.23	Right Cuneus	18	38	-18	104	0	
1.24	...	18	35	-23	104	-8	
1.25	...	19	33	-28	92	22	
1.26	...	19	28	-9	95	19	
1.27	...	18	27	-2	97	10	
1.28	...	17	23	-6	104	-7	
1.29	...	18	21	-10	102	7	
1.30	...	18	20	-2	105	3	
1.31	...	19	16	-19	97	21	
1.32	Right Declive	18	27	-35	91	-24	
1.33	Right Fusiform Gyrus	18	21	-25	98	-21	
1.34	Right Inferior Occipital Gyrus	18	37	-35	96	-8	
1.35	...	18	35	-46	85	-19	
1.36	...	18	33	-42	91	-6	
1.37	...	17	20	-27	100	-15	
1.38	...	18	17	-49	82	-6	

Target network: Smith et al. 2 (visual - continued).

	Cluster Subdivisions	BA	Voxels	Location			Peak
				x	y	z	t
1.39	Right Lingual Gyrus	18	39	-10	100	-15	
1.40	Right Middle Occipital Gyrus	19	35	-50	75	-16	
1.41	…	18	33	-28	99	7	
1.42	…	19	29	-36	87	17	
1.43	…	18	27	-17	101	13	
1.44	…	19	27	-41	87	5	
1.45	…	19	26	-32	93	8	
1.46	…	19	14	-47	80	1	
**2**	**Bilateral Frontal Cluster**	4	256	0	30	70	6.73
2.1	Left Medial Frontal Gyrus	6	25	8	15	71	
2.2	Left Paracentral Lobule	4	17	8	43	68	
2.3	Left Postcentral Gyrus	5	24	17	47	68	
2.4	…	3	21	24	32	67	
2.5	Left Precentral Gyrus	6	29	10	30	72	
2.6	Right Medial Frontal Gyrus	6	21	-8	24	72	
2.7	Right Paracentral Lobule	4	35	-4	41	68	
2.8	Right Postcentral Gyrus	3	26	-19	41	70	
2.9	Right Precentral Gyrus	4	34	-22	27	72	
2.10	Right Superior Frontal Gyrus	6	24	-4	4	70	
**3**	**Bilateral Cuneus/Precuneus Cluster**	19	189	-9	80	39	-9.54
3.1	Left Cuneus	19	24	13	83	36	
3.2	Left Precuneus	7	20	5	79	42	
3.3	Right Cuneus	19	35	-3	84	33	
3.4	Right Precuneus	19	39	-21	83	35	
3.5	…	7	24	-11	82	43	
3.6	…	7	23	-14	76	50	
3.7	Right Superior Parietal Lobule	7	24	-25	75	47	
**4**	**Right Temporal Cluster**	22	176	-62	49	8	-9.03
4.1	Right Middle Temporal Gyrus	22	36	-67	41	3	
4.2	…	37	25	-60	64	6	
4.3	…	39	24	-53	73	13	
4.4	Right Superior Temporal Gyrus	22	32	-62	54	9	
4.5	…	21	21	-68	24	-4	
4.6	…	22	18	-65	41	14	
4.7	Right Supramarginal Gyrus	40	20	-64	49	21	
**5**	**Left Temporal Cluster**	21	113	60	53	11	-8.06
5.1	Left Middle Temporal Gyrus	22	35	63	48	3	
5.2	…	19	33	57	64	13	
5.3	Left Superior Temporal Gyrus	22	23	60	56	19	
5.4	…	22	22	64	45	15	

Target network: Smith et al. 2 (visual - continued).

	Cluster Subdivisions	BA	Voxels	Location			Peak
				x	y	z	t
**6**	**Right Parietal Cluster**	40	57	-51	28	52	-5.33
6.1	Right Inferior Parietal Lobule	40	27	-48	37	56	
6.2	Right Postcentral Gyrus	2	30	-54	22	50	
**7**	**Left Inferior Parietal Lobule**	40	50	39	59	47	-4.33
7.1	…	40	28	49	49	47	
7.2	…	40	22	39	59	47	
**8**	**Left Middle Frontal Gyrus**	8	44	35	-23	46	4.90
8.1	…	8	29	24	-36	46	
8.2	…	8	15	35	-23	46	
**9**	Left Inferior Frontal Gyrus	47	19	57	-26	-6	-5.94
**10**	Left Medial Frontal Gyrus	10	6	9	-72	9	3.61
**11**	Left Middle Frontal Gyrus	6	12	38	-3	57	3.87
**12**	…	9	11	59	-13	29	-3.57
**13**	…		7	48	-50	-19	3.57
**14**	…		5	40	-64	-8	3.32
**15**	Left Middle Occipital Gyrus	19	34	42	85	18	-5.06
**16**	Left Middle Temporal Gyrus	21	12	64	30	-4	-3.90
**17**	Left Postcentral Gyrus	2	20	50	25	53	-3.80
**18**	Left Precuneus	7	5	20	74	50	-3.17
**19**	Left Superior Temporal Gyrus	22	13	60	-1	-1	4.49
**20**	Left Transverse Temporal Gyrus	42	7	64	10	15	-3.66
**21**	Left Tuber		6	50	73	-38	3.46
**22**	Right Inferior Frontal Gyrus	47	5	-59	-27	-4	-4.12
**23**	…	9	5	-54	-7	34	3.49
**24**	Right Middle Frontal Gyrus	47	18	-50	-53	-15	4.03
**25**	…		10	-44	-11	48	3.80
**26**	…	10	9	-44	-61	-2	3.70
**27**	…	9	5	-50	-15	39	3.13
**28**	Right Middle Temporal Gyrus	21	5	-62	6	-12	-4.04
**29**	Right Precentral Gyrus	43	32	-66	9	19	-5.14
**30**	…	6	9	-42	21	66	-3.43
**31**	Right Superior Temporal Gyrus	38	8	-59	-15	-24	-3.83
**32**	Right Tuber		6	-44	81	-39	3.56

Table S3: Target network: Smith et al. 3 (visual).

	Cluster Subdivisions	BA	Voxels	Location			Peak
				x	y	z	t
**1**	**Bilateral Cluster**	19	3475	-4	60	21	19.92
1.1	Left Angular Gyrus	19	17	44	82	29	
1.2	Left Cuneus	19	29	21	95	22	
1.3	...	19	27	9	86	34	
1.4	...	19	27	24	89	33	
1.5	...	19	22	14	91	25	
1.6	...	19	10	1	91	24	
1.7	Left Fusiform Gyrus	19	35	47	80	-21	
1.8	...	19	24	55	68	-19	
1.9	Left Inferior Frontal Gyrus	9	29	55	-6	36	
1.10	Left Inferior Occipital Gyrus	18	27	48	84	-7	
1.11	...	18	26	34	94	-20	
1.12	...	18	25	41	90	-13	
1.13	Left Inferior Parietal Lobule	40	38	53	53	46	
1.14	...	39	33	49	66	44	
1.15	...	40	27	38	51	57	
1.16	...	40	21	44	43	57	
1.17	...	40	18	58	41	39	
1.18	Left Inferior Temporal Gyrus	37	40	60	56	-10	
1.19	...	37	16	56	67	-2	
1.20	...	20	14	61	2	-33	
1.21	Left Lingual Gyrus	18	26	27	100	-10	
1.22	Left Middle Frontal Gyrus	6	25	36	3	65	
1.23	Left Middle Occipital Gyrus	18	38	11	98	12	
1.24	...	19	33	27	98	10	
1.25	...	18	32	37	92	9	
1.26	...	19	32	41	91	2	
1.27	...	19	28	54	75	-1	
1.28	...	19	24	47	81	6	
1.29	...	18	22	39	95	-6	
1.30	...	19	18	51	76	-11	
1.31	...	18	15	21	101	1	
1.32	Left Middle Temporal Gyrus	21	33	65	32	-2	
1.33	...	39	33	57	63	8	
1.34	...	21	29	64	17	-9	
1.35	...	39	28	49	76	20	
1.36	...	21	25	60	-4	-22	
1.37	...	39	17	50	75	9	
1.38	Left Postcentral Gyrus	1	29	55	21	50	

Target network: Smith et al. 3 (visual - continued).

	Cluster Subdivisions	BA	Voxels	Location x	y	z	Peak t
1.39	...	2	22	62	20	32	
1.40	...	2	16	52	30	56	
1.41	Left Precentral Gyrus	6	26	47	2	55	
1.42	...	4	26	40	21	67	
1.43	...	6	18	59	18	42	
1.44	Left Precuneus	7	38	23	79	42	
1.45	...	7	37	0	76	47	
1.46	...	39	32	45	76	36	
1.47	...	19	24	37	79	43	
1.48	...	7	23	11	80	41	
1.49	...	7	22	17	69	51	
1.50	...	7	11	2	67	58	
1.51	Left Superior Occipital Gyrus	19	34	34	90	20	
1.52	...	19	24	35	87	31	
1.53	Left Superior Parietal Lobule	7	38	30	59	56	
1.54	...	7	37	41	63	50	
1.55	...	7	30	27	60	65	
1.56	...	7	23	20	67	57	
1.57	...	7	22	37	73	49	
1.58	...	7	18	25	70	46	
1.59	Left Superior Temporal Gyrus	22	38	63	47	5	
1.60	...	22	29	59	58	18	
1.61	...	39	18	55	65	28	
1.62	Left Tuber		21	44	83	-41	
1.63	...		19	50	71	-32	
1.64	Right Angular Gyrus	39	22	-51	66	36	
1.65	Right Cuneus	18	37	-14	96	14	
1.66	...	19	36	-15	89	28	
1.67	...	19	26	-28	90	22	
1.68	...	18	23	-24	96	-4	
1.69	Right Declive		33	-40	86	-30	
1.70	...	19	28	-51	73	-25	
1.71	...		26	-56	64	-26	
1.72	Right Inferior Frontal Gyrus	44	40	-60	-5	23	
1.73	Right Inferior Occipital Gyrus	18	29	-42	89	-16	
1.74	...	18	25	-33	94	-14	
1.75	Right Inferior Parietal Lobule	40	29	-60	48	39	
1.76	...	40	28	-38	56	54	
1.77	...	40	24	-51	44	54	
1.78	...	40	23	-51	64	46	

Target network: Smith et al. 3 (visual - continued).

	Cluster Subdivisions	BA	Voxels	Location			Peak
				x	y	z	t
1.79	. . .	40	21	-51	53	53	
1.80	. . .	40	18	-52	55	44	
1.81	. . .	40	17	-62	42	46	
1.82	. . .	40	9	-64	43	27	
1.83	Right Inferior Temporal Gyrus	37	32	-63	53	-15	
1.84	Right Middle Frontal Gyrus	8	40	-53	-7	42	
1.85	. . .	6	15	-33	1	65	
1.86	Right Middle Occipital Gyrus	19	37	-48	82	-3	
1.87	. . .	18	34	-42	88	-4	
1.88	. . .	18	30	-33	94	0	
1.89	. . .	19	28	-54	71	-12	
1.90	. . .	37	26	-53	70	2	
1.91	. . .	19	24	-28	94	13	
1.92	. . .	19	22	-36	91	10	
1.93	. . .	19	21	-41	86	10	
1.94	. . .	18	19	-49	79	-15	
1.95	Right Middle Temporal Gyrus	37	40	-59	66	-3	
1.96	. . .	21	34	-64	1	-18	
1.97	. . .	21	32	-67	14	-7	
1.98	. . .	22	31	-57	63	14	
1.99	. . .	37	28	-61	57	-5	
1.100	. . .	21	27	-67	36	-4	
1.101	. . .	39	26	-48	74	15	
1.102	. . .	39	25	-51	78	9	
1.103	. . .	39	20	-42	76	23	
1.104	. . .		15	-63	-3	-31	
1.105	Right Postcentral Gyrus	3	38	-60	25	43	
1.106	. . .	5	34	-37	50	62	
1.107	. . .	2	32	-48	33	59	
1.108	. . .	1	28	-55	21	50	
1.109	. . .	3	25	-65	18	32	
1.110	. . .	43	21	-65	18	22	
1.111	. . .	3	20	-47	20	61	
1.112	. . .	2	14	-38	41	69	
1.113	Right Precentral Gyrus	6	37	-38	9	65	
1.114	. . .	6	28	-48	3	54	
1.115	Right Precuneus	19	38	-43	76	36	
1.116	. . .	19	28	-30	85	32	
1.117	. . .	7	21	-19	79	44	
1.118	. . .	7	21	-14	73	51	

Target network: Smith et al. 3 (visual - continued).

	Cluster Subdivisions	BA	Voxels	Location			Peak
				x	y	z	t
1.119	…	19	21	-22	83	35	
1.120	Right Pyramis		29	-46	79	-41	
1.121	Right Superior Occipital Gyrus	19	30	-38	84	24	
1.122	Right Superior Parietal Lobule	7	40	-22	62	61	
1.123	…	7	33	-28	66	50	
1.124	…	7	30	-38	71	47	
1.125	…	7	26	-27	75	44	
1.126	…	7	23	-32	64	59	
1.127	…	7	14	-14	65	61	
1.128	Right Superior Temporal Gyrus	22	40	-64	47	8	
1.129	…	22	22	-69	25	1	
1.130	Right Supramarginal Gyrus	39	35	-57	63	31	
1.131	…	40	9	-62	50	22	
**2**	**Bilateral Frontal/Central Cluster**	4	307	-2	20	67	-5.97
2.1	Left Medial Frontal Gyrus	6	35	8	13	69	
2.2	Left Middle Frontal Gyrus	6	34	21	15	67	
2.3	Left Postcentral Gyrus	3	37	23	32	68	
2.4	…	3	23	15	41	69	
2.5	Right Paracentral Lobule	5	20	-2	50	66	
2.6	Right Postcentral Gyrus	3	29	-23	38	68	
2.7	Right Precentral Gyrus	4	25	-28	26	67	
2.8	…	6	12	-13	25	70	
2.9	Right Superior Frontal Gyrus	6	40	-20	-9	60	
2.10	…	6	29	-16	14	69	
2.11	…	6	23	-2	6	68	
**3**	**Bilateral Cuneus Cluster**	18	51	1	106	4	-5.13
3.1	Left Cuneus		19	11	110	1	
3.2	Right Cuneus	18	32	-4	104	7	
**4**	**Left Middle Frontal Gyrus**	9	44	28	-30	44	-4.60
4.1	…	9	25	46	-17	39	
4.2	…	9	19	28	-30	44	
**5**	Left Inferior Frontal Gyrus	47	11	55	-34	-18	3.17
**6**	…	46	9	46	-36	17	-3.94
**7**	Left Middle Frontal Gyrus	10	21	41	-50	12	-4.07
**8**	…	10	8	26	-61	29	3.59
**9**	…		6	36	-69	-11	2.81
**10**	…	8	5	33	-14	50	-2.77
**11**	Left Precentral Gyrus		6	15	36	80	2.80
**12**	Left Superior Temporal Gyrus	22	36	61	-8	-3	-5.26
**13**	Right Middle Frontal Gyrus	10	7	-47	-56	2	-2.63

Target network: Smith et al. 3 (visual - continued).

	Cluster Subdivisions	BA	Voxels	Location			Peak
				x	y	z	t
**14**	Right Precentral Gyrus	9	36	-43	-23	41	-4.25
**15**	Right Superior Frontal Gyrus	10	10	-38	-55	18	-3.17
**16**	...	6	5	-8	-24	65	2.89
**17**	Right Superior Temporal Gyrus	22	28	-62	-9	-1	-4.53

Table S4: Target network: Smith et al. 4 (default mode network).

	Cluster Subdivisions	BA	Voxels	Location			Peak
				x	y	z	t
**1**	**Bilateral Frontal/Temporal Cluster**	22	1587	-1	-13	33	-11.25
1.1	Left Inferior Frontal Gyrus	44	38	58	-12	19	
1.2	...	47	33	53	-33	-7	
1.3	...	47	30	58	-19	-4	
1.4	...	10	25	50	-44	0	
1.5	...	47	22	50	-47	-12	
1.6	Left Medial Frontal Gyrus	6	29	4	31	69	
1.7	...	6	17	11	0	66	
1.8	Left Middle Frontal Gyrus	10	37	44	-51	6	
1.9	...	6	37	26	-10	59	
1.10	...	9	36	54	-17	31	
1.11	...	46	33	43	-42	19	
1.12	...	9	32	44	-33	31	
1.13	...	46	28	49	-31	21	
1.14	...	10	26	46	-53	-12	
1.15	...	6	24	25	10	65	
1.16	...	9	15	50	-5	42	
1.17	Left Postcentral Gyrus	5	27	31	45	71	
1.18	...	3	21	16	40	74	
1.19	Left Precentral Gyrus	6	37	49	2	47	
1.20	...	4	33	41	15	57	
1.21	...	44	22	58	-5	10	
1.22	...	6	22	17	22	75	
1.23	...	4	21	26	26	75	
1.24	...	4	20	33	29	66	
1.25	Left Superior Frontal Gyrus	6	29	18	-8	63	
1.26	...	10	23	35	-54	19	
1.27	...	6	20	13	10	68	
1.28	...	10	19	37	-49	26	
1.29	Left Superior Temporal Gyrus	22	32	60	-10	1	
1.30	Right Inferior Frontal Gyrus	45	38	-58	-20	20	
1.31	...	46	28	-48	-45	16	
1.32	...	47	27	-59	-19	-4	
1.33	...	44	27	-60	-7	25	
1.34	...	47	26	-53	-40	-12	
1.35	...	45	25	-54	-31	10	
1.36	...	46	24	-51	-46	1	
1.37	...	10	23	-42	-61	3	

Target network: Smith et al. 4 (default mode network - continued).

	Cluster Subdivisions	BA	Voxels	Location			Peak
				x	y	z	t
1.38	Right Middle Frontal Gyrus	10	38	-48	-55	-9	
1.39	…	9	35	-46	-30	36	
1.40	…	9	33	-56	-7	37	
1.41	…	6	32	-41	5	59	
1.42	…	46	32	-42	-42	29	
1.43	…	6	27	-50	2	46	
1.44	…	10	24	-41	-57	15	
1.45	…	6	24	-31	12	65	
1.46	…	6	24	-20	3	65	
1.47	…	10	20	-47	-50	7	
1.48	…	6	14	-39	-5	55	
1.49	…	46	13	-50	-37	19	
1.50	Right Postcentral Gyrus	3	26	-39	35	69	
1.51	…		16	-23	38	78	
1.52	Right Precentral Gyrus	4	39	-45	22	63	
1.53	…	4	32	-23	26	75	
1.54	Right Superior Frontal Gyrus	6	36	-8	11	69	
1.55	…	6	28	-4	-4	64	
1.56	…	6	27	-25	-7	61	
1.57	…	10	22	-36	-55	21	
1.58	Right Superior Temporal Gyrus	22	39	-61	-9	3	
**2**	**Bilateral Frontal Cluster**	10	560	-4	-60	18	8.29
2.1	Left Medial Frontal Gyrus	10	31	1	-67	11	
2.2	…	10	13	9	-64	15	
2.3	Left Middle Frontal Gyrus	10	28	22	-69	11	
2.4	Left Superior Frontal Gyrus	10	39	9	-69	2	
2.5	…	10	32	20	-68	-2	
2.6	…	10	32	13	-65	24	
2.7	…	10	20	15	-65	10	
2.8	Right Medial Frontal Gyrus	10	36	-1	-67	-5	
2.9	…	10	31	-8	-71	6	
2.10	…	11	28	-3	-66	-17	
2.11	…	10	26	-2	-67	25	
2.12	…		16	-5	-72	20	
2.13	Right Middle Frontal Gyrus	8	26	-27	-35	47	
2.14	Right Superior Frontal Gyrus	9	34	-25	-43	42	
2.15	…	6	33	-16	-31	60	
2.16	…	10	29	-22	-67	-1	
2.17	…	9	28	-7	-62	36	

Target network: Smith et al. 4 (default mode network - continued).

	Cluster Subdivisions	BA	Voxels	Location			Peak
				x	y	z	t
2.18	...	10	28	-19	-70	11	
2.19	...	9	27	-20	-54	36	
2.20	...	8	23	-26	-31	55	
**3**	**Right Temporal Cluster**	39	549	-57	43	9	17.41
3.1	Right Angular Gyrus	19	29	-43	76	32	
3.2	Right Fusiform Gyrus	20	21	-64	4	-32	
3.3	Right Inferior Parietal Lobule	40	39	-53	57	39	
3.4	...	40	24	-41	66	47	
3.5	...	40	17	-46	65	40	
3.6	Right Inferior Temporal Gyrus	20	34	-67	19	-23	
3.7	Right Middle Temporal Gyrus	21	40	-64	1	-21	
3.8	...	39	35	-49	73	24	
3.9	...	21	33	-66	11	-10	
3.10	...	21	31	-60	-10	-34	
3.11	...	39	26	-54	69	12	
3.12	...	39	25	-55	66	28	
3.13	...	21	20	-68	32	-9	
3.14	...	39	19	-59	61	9	
3.15	...	21	12	-70	27	-5	
3.16	Right Precuneus	19	26	-44	73	42	
3.17	Right Superior Temporal Gyrus	22	33	-66	41	4	
3.18	...	22	25	-63	47	19	
3.19	...	22	20	-62	57	14	
3.20	...	39	19	-57	58	28	
3.21	Right Supramarginal Gyrus	22	21	-61	57	22	
**4**	**Bilateral Precuneus/Occipital Cluster**	7	481	-1	83	35	10.04
4.1	Left Cuneus	18	38	25	97	20	
4.2	...	19	33	19	93	34	
4.3	...	19	30	13	99	22	
4.4	...	19	23	5	85	33	
4.5	...	19	12	2	93	24	
4.6	Left Middle Occipital Gyrus	19	25	33	93	11	
4.7	Left Precuneus	7	39	6	66	55	
4.8	...	7	34	6	76	49	
4.9	Right Cuneus	19	36	-19	88	36	
4.10	Right Middle Occipital Gyrus	19	25	-28	91	15	
4.11	Right Precuneus	7	35	-1	81	40	
4.12	...	7	35	-9	80	42	
4.13	...	7	27	-5	60	61	

Target network: Smith et al. 4 (default mode network - continued).

	Cluster Subdivisions	BA	Voxels	Location			Peak
				x	y	z	t
4.14	…	7	23	-27	81	45	
4.15	…	7	18	-15	74	50	
4.16	Right Superior Occipital Gyrus	19	33	-35	91	20	
4.17	Right Superior Parietal Lobule	7	15	-16	67	54	
**5**	**Left Parietal/Temporal Cluster**	39	412	46	69	30	17.03
5.1	Left Angular Gyrus	39	38	49	72	31	
5.2	Left Inferior Parietal Lobule	39	25	46	68	41	
5.3	…	40	20	46	60	42	
5.4	Left Middle Temporal Gyrus	39	23	52	71	14	
5.5	…	39	21	43	79	20	
5.6	…	19	21	57	65	14	
5.7	…	39	19	50	77	21	
5.8	Left Precuneus	19	30	35	81	39	
5.9	…	19	21	31	73	39	
5.10	Left Superior Occipital Gyrus	19	39	38	83	30	
5.11	Left Superior Parietal Lobule	7	29	38	67	50	
5.12	…	7	24	36	75	47	
5.13	Left Superior Temporal Gyrus	39	28	56	64	27	
5.14	…	22	25	60	54	17	
5.15	…	22	18	63	47	15	
5.16	Left Supramarginal Gyrus	40	31	57	56	30	
**6**	**Left Temporal Cluster**	21	225	63	15	-17	10.08
6.1	Left Inferior Temporal Gyrus	20	34	64	10	-27	
6.2	Left Middle Temporal Gyrus	21	34	64	6	-17	
6.3	…	22	32	66	32	1	
6.4	…	21	30	66	20	-12	
6.5	…	21	29	60	-3	-33	
6.6	…	21	24	64	38	-10	
6.7	…	21	22	61	-7	-24	
6.8	…	21	20	64	26	-18	
**7**	**Right Parietal Cluster**	2	177	-61	33	39	-8.01
7.1	Right Inferior Parietal Lobule	40	40	-55	44	51	
7.2	…	40	37	-65	25	23	
7.3	…	40	27	-67	34	32	
7.4	…	40	21	-63	37	43	
7.5	Right Postcentral Gyrus	2	28	-58	30	49	
7.6	…	2	24	-65	26	39	
**8**	**Left Occipital Cluster**		151	26	101	-16	5.32
8.1	Left Cuneus		32	13	110	-5	

Target network: Smith et al. 4 (default mode network - continued).

	Cluster Subdivisions	BA	Voxels	Location			Peak
				x	y	z	t
8.2	Left Inferior Occipital Gyrus	18	28	33	99	-13	
8.3	...	18	17	36	94	-25	
8.4	Left Lingual Gyrus		28	15	108	-17	
8.5	Left Middle Occipital Gyrus	18	22	28	106	-3	
8.6	Left Pyramis		24	42	84	-42	
**9**	**Right Occipital Cluster**	18	81	-26	100	-8	5.51
9.1	Right Cuneus	18	33	-19	105	-4	
9.2	Right Inferior Occipital Gyrus	18	28	-34	97	-9	
9.3	...	17	20	-28	101	-16	
**10**	**Left Superior Frontal Gyrus**	8	74	13	-47	46	3.96
10.1	...	8	33	24	-37	49	
10.2	...	9	27	21	-45	39	
10.3	...	8	14	13	-47	46	
**11**	**Left Inferior Parietal Lobule**	40	73	54	46	51	-4.94
11.1	...	40	37	61	37	45	
11.2	...	40	36	54	46	51	
**12**	Left Inferior Parietal Lobule	40	12	66	34	31	-3.29
**13**	Left Middle Frontal Gyrus	10	6	39	-65	-4	-2.88
**14**	Left Middle Occipital Gyrus	37	38	56	63	-11	-5.06
**15**	Left Postcentral Gyrus	40	6	64	21	19	-3.81
**16**	Left Superior Frontal Gyrus	8	11	37	-18	54	3.46
**17**	...	6	10	9	-37	63	3.42
**18**	...		7	36	17	70	2.59
**19**	Left Superior Parietal Lobule	7	8	16	65	61	-3.52
**20**	Right Declive	19	7	-51	71	-16	-3.12
**21**	Right Inferior Temporal Gyrus	20	19	-61	57	-18	-4.76
**22**	Right Middle Frontal Gyrus	8	5	-46	-17	50	3.06
**23**	Right Superior Frontal Gyrus	8	25	1	-32	55	-4.70
**24**	...		5	-36	-70	-5	-3.53
**25**	Right Tuber		24	-47	78	-42	4.55
**26**	...		7	-36	90	-39	3.06

Table S5: Target network: Smith et al. 5 (cerebellum).

	**Cluster Subdivisions**	**BA**	**Voxels**	**Location**			**Peak**
				**x**	**y**	**z**	**t**
**1**	**Right Occipital Cluster**	18	55	-4	100	-5	6.61
1.1	Right Cuneus	18	19	-3	99	3	
1.2	Right Lingual Gyrus	18	36	-6	101	-9	
**2**	Left Fusiform Gyrus	19	5	47	79	-22	4.00
**3**	Left Inferior Temporal Gyrus	20	5	58	60	-21	4.88
**4**	...	21	5	63	4	-20	4.11
**5**	Left Medial Frontal Gyrus	10	27	11	-68	12	4.41
**6**	Left Postcentral Gyrus	7	9	14	58	68	4.55
**7**	Left Precentral Gyrus	4	9	55	15	38	-4.57
**8**	Left Precuneus	19	21	42	77	35	4.79
**9**	Left Superior Parietal Lobule	7	26	9	72	54	4.85
**10**	...	7	5	30	77	47	4.38
**11**	Left Superior Temporal Gyrus	22	33	61	-10	-2	6.42
**12**	Right Cuneus	18	7	-4	96	19	3.87
**13**	...	19	5	-32	89	26	-4.13
**14**	Right Fusiform Gyrus	20	13	-58	62	-19	5.63
**15**	...	19	10	-47	78	-20	5.24
**16**	Right Inferior Frontal Gyrus	9	7	-52	-17	33	-4.86
**17**	Right Middle Temporal Gyrus	39	8	-46	77	12	5.37
**18**	Right Postcentral Gyrus	5	7	-8	49	73	4.47
**19**	...	42	5	-66	23	14	4.07
**20**	Right Superior Parietal Lobule	7	17	-14	64	62	4.62
**21**	Right Superior Temporal Gyrus	22	11	-61	-8	-1	4.55

Table S6: Target network: Smith et al. 6 (sensorimotor).

	Cluster Subdivisions	BA	Voxels	Location			Peak
				x	y	z	t
**1**	**Bilateral Frontal/Parietal Cluster**	4	2908	-3	9	48	14.09
1.1	Left Inferior Frontal Gyrus	9	30	57	-4	30	
1.2	. . .	9	20	62	-7	23	
1.3	Left Inferior Parietal Lobule	40	32	56	35	43	
1.4	. . .	40	31	41	52	54	
1.5	. . .	40	25	52	38	56	
1.6	. . .	40	23	32	54	59	
1.7	. . .	40	23	47	41	50	
1.8	Left Middle Frontal Gyrus	9	33	39	-28	40	
1.9	. . .	6	30	27	-19	62	
1.10	. . .	6	29	40	-8	59	
1.11	. . .	8	28	48	-6	43	
1.12	. . .	8	22	40	-21	51	
1.13	. . .	6	20	37	1	60	
1.14	. . .	8	19	45	-18	43	
1.15	. . .	6	18	25	-7	66	
1.16	Left Paracentral Lobule	4	37	7	38	68	
1.17	Left Postcentral Gyrus	5	38	21	47	69	
1.18	. . .	3	37	62	12	27	
1.19	. . .	1	29	53	21	46	
1.20	. . .	3	28	38	34	62	
1.21	. . .	2	26	60	24	32	
1.22	. . .	3	25	43	23	58	
1.23	. . .	4	22	19	35	72	
1.24	. . .	5	20	35	44	61	
1.25	. . .	3	13	60	25	47	
1.26	Left Precentral Gyrus	6	38	62	2	16	
1.27	. . .	4	38	27	33	66	
1.28	. . .	6	36	47	5	51	
1.29	. . .	6	33	24	19	69	
1.30	. . .	6	29	54	8	40	
1.31	. . .	6	27	31	13	62	
1.32	. . .	4	26	40	15	56	
1.33	. . .	6	25	60	7	35	
1.34	. . .	44	8	59	-9	9	
1.35	Left Precuneus	7	17	23	60	53	
1.36	Left Superior Frontal Gyrus	8	39	10	-45	53	
1.37	. . .	6	39	19	3	69	
1.38	. . .	6	39	11	19	71	

Target network: Smith et al. 6 (sensorimotor - continued).

	Cluster Subdivisions	BA	Voxels	Location			Peak
				x	y	z	t
1.39	...	8	30	31	-29	54	
1.40	...	8	28	16	-30	59	
1.41	...	6	25	12	-15	68	
1.42	...	8	17	27	-39	47	
1.43	...	6	17	5	6	68	
1.44	Left Superior Parietal Lobule	7	29	17	56	63	
1.45	...	7	25	24	69	55	
1.46	...	7	19	32	58	50	
1.47	Left Superior Temporal Gyrus	22	33	62	0	4	
1.48	...	22	31	64	16	4	
1.49	...	42	30	64	26	14	
1.50	Right Inferior Frontal Gyrus	9	28	-61	-3	26	
1.51	...	9	21	-59	-15	30	
1.52	...	45	20	-63	-10	20	
1.53	Right Inferior Parietal Lobule	40	31	-47	44	58	
1.54	...	40	29	-53	45	50	
1.55	...	40	26	-39	56	54	
1.56	...	40	22	-65	27	30	
1.57	Right Medial Frontal Gyrus	6	31	-3	13	68	
1.58	...	9	29	-6	-55	42	
1.59	...	6	22	-4	26	69	
1.60	Right Middle Frontal Gyrus	9	33	-39	-31	42	
1.61	...	8	30	-31	-33	53	
1.62	...	6	30	-50	-8	47	
1.63	...	6	28	-24	2	66	
1.64	...	6	28	-22	-10	69	
1.65	...	6	28	-38	-11	59	
1.66	...	8	21	-47	-19	45	
1.67	...	6	21	-36	3	59	
1.68	...	6	20	-43	-4	55	
1.69	Right Postcentral Gyrus	40	40	-67	29	18	
1.70	...	2	38	-57	31	46	
1.71	...	5	34	-9	46	67	
1.72	...	3	33	-37	38	63	
1.73	...	3	28	-51	15	51	
1.74	...	43	26	-66	13	19	
1.75	...	3	26	-62	23	39	
1.76	...	3	25	-45	24	57	
1.77	...	40	17	-56	35	56	
1.78	Right Precentral Gyrus	4	38	-30	29	67	

Target network: Smith et al. 6 (sensorimotor - continued).

	Cluster Subdivisions	BA	Voxels	Location x	y	z	Peak t
1.79	...	6	31	-61	8	34	
1.80	...	6	30	-42	10	55	
1.81	...	6	28	-66	2	14	
1.82	...	6	28	-22	18	69	
1.83	...	4	27	-36	20	63	
1.84	...	4	24	-13	31	71	
1.85	...	6	21	-54	0	39	
1.86	...	4	21	-56	9	46	
1.87	...	6	19	-61	16	40	
1.88	...	4	17	-19	27	70	
1.89	...	4	16	-64	8	27	
1.90	Right Superior Frontal Gyrus	8	35	-31	-22	59	
1.91	...	8	32	-10	-48	51	
1.92	...	10	31	-21	-59	28	
1.93	...	9	27	-33	-42	38	
1.94	...	6	26	-11	-25	61	
1.95	...	8	25	-21	-42	51	
1.96	...	6	25	-12	-15	68	
1.97	...	8	24	-17	-31	58	
1.98	...	9	22	-22	-51	39	
1.99	...	6	21	-12	7	70	
1.100	...	6	20	-21	11	71	
1.101	Right Superior Parietal Lobule	7	38	-21	64	57	
1.102	...	7	33	-33	51	62	
1.103	...	7	32	-30	65	54	
1.104	...	5	20	-24	45	65	
1.105	Right Superior Temporal Gyrus	22	37	-62	-7	4	
1.106	...	22	34	-64	5	2	
1.107	...	42	25	-67	20	9	
**2**	**Left Temporal/Occipital Cluster**	19	368	52	65	12	-7.25
2.1	Left Cuneus	19	19	30	84	31	
2.2	Left Inferior Temporal Gyrus	37	31	59	63	-11	
2.3	Left Middle Occipital Gyrus	19	28	30	90	18	
2.4	...	19	18	40	87	9	
2.5	Left Middle Temporal Gyrus	39	38	50	75	12	
2.6	...	21	32	61	60	5	
2.7	...	21	24	65	43	-7	
2.8	...	39	22	50	71	28	
2.9	...	21	20	63	54	-2	
2.10	...	21	17	65	33	-14	

Target network: Smith et al. 6 (sensorimotor - continued).

	Cluster Subdivisions	BA	Voxels	Location			Peak
				x	y	z	t
2.11	Left Superior Occipital Gyrus	19	27	41	80	26	
2.12	Left Superior Temporal Gyrus	39	39	57	65	18	
2.13	Left Supramarginal Gyrus	40	35	63	49	21	
2.14	...	40	18	59	56	32	
**3**	**Right Temporal Cluster**	22	234	-59	50	5	-6.94
3.1	Right Inferior Temporal Gyrus	21	29	-65	10	-19	
3.2	Right Middle Temporal Gyrus	39	27	-50	71	20	
3.3	...	21	26	-62	55	3	
3.4	...	21	25	-65	44	-10	
3.5	...	39	24	-41	79	15	
3.6	...	21	23	-68	30	-11	
3.7	Right Superior Temporal Gyrus	39	27	-56	64	21	
3.8	...	22	24	-64	51	13	
3.9	Right Supramarginal Gyrus	40	29	-62	52	22	
**4**	**Left Frontal Cluster**	45	172	54	-30	-2	-6.90
4.1	Left Inferior Frontal Gyrus	47	38	55	-30	-12	
4.2	...	47	31	50	-45	-13	
4.3	...	46	31	53	-28	13	
4.4	...	45	27	59	-20	8	
4.5	...	47	26	58	-21	-3	
4.6	Left Middle Frontal Gyrus	10	19	49	-42	-1	
**5**	**Right Frontal Cluster**	45	151	-56	-28	-3	-7.05
5.1	Right Inferior Frontal Gyrus	45	39	-56	-32	2	
5.2	...	45	33	-57	-23	14	
5.3	...	47	21	-53	-43	-14	
5.4	...	47	20	-56	-32	-9	
5.5	Right Superior Temporal Gyrus	38	38	-58	-21	-14	
**6**	**Right Frontal Cluster**	10	87	-39	-61	12	4.77
6.1	Right Middle Frontal Gyrus	46	28	-49	-48	19	
6.2	...	10	21	-46	-59	9	
6.3	Right Superior Frontal Gyrus	10	38	-28	-73	9	
**7**	**Bilateral Cuneus/Precuneus Cluster**	19	66	-3	84	31	-4.99
7.1	Left Cuneus	18	23	1	89	22	
7.2	Left Precuneus	7	22	1	82	38	
7.3	Right Cuneus	19	21	-12	84	36	
**8**	**Left Temporal Cluster**	21	55	60	0	-23	
8.1	Left Inferior Temporal Gyrus	21	19	61	6	-18	
8.2	Left Middle Temporal Gyrus	21	36	60	-4	-26	
**9**	**Right Inferior Occipital Gyrus**	18	55	-40	91	-17	4.25
9.1	...	17	28	-30	99	-15	

Target network: Smith et al. 6 (sensorimotor - continued).

	Cluster Subdivisions	BA	Voxels	Location			Peak
				x	y	z	t
9.2	...	18	27	-40	91	-17	
**10**	**Left Occipital Cluster**	19	47	18	101	20	4.23
10.1	Left Cuneus	19	18	14	97	31	
10.2	Left Middle Occipital Gyrus	19	29	21	105	14	
**11**	**Left Middle Frontal Gyrus**	46	44	43	-58	13	3.86
11.1	...	46	26	42	-48	22	
11.2	...	10	18	43	-58	13	
**12**	**Right Middle Occipital Gyrus**	19	43	-42	91	3	4.26
12.1	...	19	22	-32	95	16	
12.2	...	19	21	-42	91	3	
**13**	Left Cuneus	17	8	4	101	-2	-4.16
**14**	Left Declive		13	41	89	-26	3.86
**15**	Left Inferior Frontal Gyrus	10	5	40	-52	4	-3.60
**16**	Left Inferior Occipital Gyrus	18	24	27	100	-23	3.63
**17**	Left Inferior Parietal Lobule	40	6	51	57	45	2.76
**18**	Left Lingual Gyrus		6	16	110	-16	3.07
**19**	Left Medial Frontal Gyrus		6	7	-73	1	3.65
**20**	...		5	4	31	80	-2.84
**21**	Left Superior Frontal Gyrus	10	28	25	-64	2	-5.27
**22**	...	10	14	17	-70	22	3.23
**23**	...	9	8	31	-49	36	-3.30
**24**	Left Superior Parietal Lobule	7	5	28	60	69	-2.71
**25**	Right Cuneus	17	9	-11	102	-1	-3.20
**26**	...	18	7	-12	103	13	2.90
**27**	...	19	5	-17	98	26	3.54
**28**	Right Middle Frontal Gyrus	10	10	-32	-61	10	-3.35
**29**	...	10	8	-42	-56	2	-3.83
**30**	Right Middle Occipital Gyrus	19	32	-53	71	-15	4.22
**31**	Right Postcentral Gyrus	7	32	-9	64	65	-4.27
**32**	Right Precuneus	7	16	-28	84	39	3.56
**33**	Right Superior Frontal Gyrus	10	10	-18	-66	10	-2.99

Table S7: Target network: Smith et al. 7 (auditory).

	Cluster Subdivisions	BA	Voxels	Location			Peak
				x	y	z	t
**1**	**Mostly Left Hemisphere Cluster**	22	2537	30	-3	29	18.56
1.1	Left Inferior Frontal Gyrus	44	39	60	-12	15	
1.2	...	46	31	51	-38	3	
1.3	...	46	28	49	-29	18	
1.4	...	47	26	52	-46	-14	
1.5	...	47	23	53	-33	-14	
1.6	...	45	22	57	-9	26	
1.7	...	45	21	55	-26	5	
1.8	...	47	20	58	-23	-4	
1.9	...	47	20	56	-33	-7	
1.10	...	45	11	55	-19	20	
1.11	Left Inferior Parietal Lobule	40	38	63	43	32	
1.12	...	40	29	63	33	36	
1.13	...	40	27	65	25	30	
1.14	...	40	9	60	41	51	
1.15	Left Inferior Temporal Gyrus	37	28	54	69	-1	
1.16	Left Medial Frontal Gyrus		26	2	-56	49	
1.17	...	9	25	5	-50	43	
1.18	Left Middle Frontal Gyrus	9	33	35	-29	37	
1.19	...	9	30	44	-14	40	
1.20	...	9	28	25	-39	40	
1.21	...	47	26	48	-48	-4	
1.22	...	6	25	32	-3	59	
1.23	...	6	19	34	-11	52	
1.24	...	9	19	37	-21	44	
1.25	Left Middle Temporal Gyrus	21	40	62	55	3	
1.26	...	21	35	63	6	-15	
1.27	...	21	33	65	21	-10	
1.28	...	37	31	58	61	-3	
1.29	...	19	29	45	85	21	
1.30	...	21	23	66	35	-5	
1.31	...	21	22	65	44	-3	
1.32	...	39	22	52	74	8	
1.33	...	21	21	59	-8	-28	
1.34	...	19	20	52	76	17	
1.35	Left Postcentral Gyrus	2	31	54	24	55	
1.36	...	1	30	46	35	64	
1.37	...	3	30	63	11	23	
1.38	...	40	28	66	30	20	

Target network: Smith et al. 7 (auditory - continued).

	Cluster Subdivisions	BA	Voxels	Location			Peak
				x	y	z	t
1.39	…	1	22	63	19	31	
1.40	…	3	21	20	38	71	
1.41	…	43	10	66	12	15	
1.42	Left Precentral Gyrus	4	34	51	8	51	
1.43	…	6	32	15	19	73	
1.44	…	6	31	62	2	12	
1.45	…	6	28	58	16	43	
1.46	…	6	26	37	20	68	
1.47	…	4	25	27	30	69	
1.48	…	6	24	58	9	35	
1.49	…	6	20	59	1	27	
1.50	…	6	18	39	8	60	
1.51	Left Superior Frontal Gyrus	8	38	15	-43	48	
1.52	…	9	38	11	-62	36	
1.53	…	8	37	21	-29	52	
1.54	…	10	25	27	-45	31	
1.55	…	8	24	1	-37	61	
1.56	…	6	23	4	6	68	
1.57	…	6	22	26	-16	57	
1.58	…	8	20	13	-40	58	
1.59	…	6	19	16	13	71	
1.60	…	10	16	22	-56	28	
1.61	…	6	12	19	3	68	
1.62	Left Superior Occipital Gyrus	19	13	36	91	27	
1.63	Left Superior Temporal Gyrus	22	35	64	13	3	
1.64	…	22	35	63	44	15	
1.65	…	22	33	61	58	14	
1.66	…	22	32	61	-9	-3	
1.67	…	38	31	56	-22	-22	
1.68	…	44	29	60	-13	4	
1.69	…	22	28	63	3	-3	
1.70	…	42	28	67	21	11	
1.71	…	38	27	58	-10	-13	
1.72	…	22	26	67	35	10	
1.73	…	21	23	66	29	2	
1.74	…	39	16	60	58	25	
1.75	Left Supramarginal Gyrus	40	21	62	49	25	
1.76	Right Medial Frontal Gyrus		32	-1	-72	20	
1.77	…	6	24	-5	19	72	
1.78	Right Middle Frontal Gyrus	8	35	-41	-16	45	

Target network: Smith et al. 7 (auditory - continued).

	Cluster Subdivisions	BA	Voxels	Location x	y	z	Peak t
1.79	...	9	35	-37	-30	38	
1.80	...	46	31	-43	-45	28	
1.81	...	9	29	-50	-22	35	
1.82	...	6	27	-31	2	59	
1.83	...	8	27	-24	-32	46	
1.84	...	9	23	-26	-41	39	
1.85	...	46	20	-49	-32	27	
1.86	...	8	17	-42	-6	49	
1.87	Right Superior Frontal Gyrus	8	39	-10	-49	54	
1.88	...	10	33	-15	-64	32	
1.89	...	9	32	-29	-49	34	
1.90	...	8	31	-15	-49	44	
1.91	...	6	28	-9	8	71	
1.92	...	8	27	-29	-19	53	
1.93	...	9	22	-8	-61	40	
1.94	...	6	18	-14	-7	65	
1.95	...	6	18	-3	-21	67	
1.96	...	8	18	-17	-34	60	
1.97	...	6	16	-33	-9	57	
1.98	...	8	15	-25	-27	55	
**2**	**Right Hemisphere Cluster**	42	1369	-59	6	10	21.84
2.1	Right Inferior Frontal Gyrus	44	38	-61	-13	19	
2.2	...	45	36	-60	-22	3	
2.3	...	46	35	-56	-27	15	
2.4	...	47	35	-56	-34	0	
2.5	...	47	28	-55	-34	-15	
2.6	...	47	24	-53	-45	-15	
2.7	...	47	24	-59	-25	-8	
2.8	...	9	23	-59	-8	29	
2.9	...	46	20	-51	-41	6	
2.10	Right Inferior Parietal Lobule	40	32	-66	41	26	
2.11	...	40	26	-66	34	35	
2.12	...	40	20	-59	40	53	
2.13	Right Inferior Temporal Gyrus	37	22	-61	61	-7	
2.14	Right Middle Frontal Gyrus	47	28	-50	-48	-1	
2.15	...		26	-49	-56	-16	
2.16	...	47	21	-53	-50	-8	
2.17	...	10	21	-42	-64	-11	
2.18	...	6	21	-56	-2	43	
2.19	Right Middle Temporal Gyrus	21	40	-60	-10	-24	

Target network: Smith et al. 7 (auditory - continued).

	Cluster Subdivisions	BA	Voxels	Location			Peak
				x	y	z	t
2.20	...	21	39	-59	62	5	
2.21	...	21	38	-63	0	-15	
2.22	...	21	29	-66	15	-14	
2.23	...	21	24	-66	48	-3	
2.24	...	39	24	-54	68	13	
2.25	...	21	23	-70	23	-7	
2.26	...	19	23	-50	78	18	
2.27	...	21	21	-69	37	-5	
2.28	...	21	17	-66	51	3	
2.29	Right Postcentral Gyrus	43	40	-66	15	19	
2.30	...	2	40	-65	23	33	
2.31	...	3	24	-47	27	63	
2.32	...	2	21	-63	30	48	
2.33	...	3	21	-51	15	58	
2.34	Right Precentral Gyrus	6	28	-50	2	54	
2.35	...	4	26	-62	5	24	
2.36	...	44	25	-64	-7	10	
2.37	...	6	23	-61	11	40	
2.38	...	6	18	-60	3	33	
2.39	...	22	17	-65	4	12	
2.40	...	4	15	-56	11	50	
2.41	Right Superior Frontal Gyrus		22	-34	-70	-13	
2.42	Right Superior Temporal Gyrus	42	40	-68	22	6	
2.43	...	42	36	-66	29	16	
2.44	...	22	35	-66	37	7	
2.45	...	22	25	-65	0	0	
2.46	...	22	24	-64	52	11	
2.47	...	38	22	-56	-23	-25	
2.48	...	22	20	-61	-8	-6	
2.49	...	22	20	-61	-12	2	
2.50	...	21	20	-66	9	-2	
2.51	...	22	19	-67	16	1	
2.52	Right Supramarginal Gyrus	40	30	-63	49	21	
**3**	**Bilateral Parietal/Occipital Cluster**	40	1223	-4	70	41	-9.60
3.1	Left Angular Gyrus	39	39	34	79	31	
3.2	...	40	28	51	64	36	
3.3	Left Cuneus	18	18	6	98	16	
3.4	Left Inferior Parietal Lobule	7	26	36	52	59	
3.5	...	40	24	43	62	44	
3.6	...	39	20	42	69	40	

Target network: Smith et al. 7 (auditory - continued).

	Cluster Subdivisions	BA	Voxels	Location			Peak
				x	y	z	t
3.7	...	40	19	46	47	50	
3.8	...	40	17	52	51	43	
3.9	Left Middle Occipital Gyrus	18	34	20	97	11	
3.10	...	19	17	35	91	8	
3.11	Left Precuneus	7	39	8	74	51	
3.12	...	7	36	14	69	50	
3.13	...	19	31	27	73	42	
3.14	...	7	28	18	65	58	
3.15	...	7	25	4	62	61	
3.16	...	19	22	3	82	37	
3.17	Left Superior Parietal Lobule	7	31	36	57	51	
3.18	...	7	23	30	65	54	
3.19	...	7	22	35	73	44	
3.20	...	7	19	25	61	61	
3.21	Right Angular Gyrus	40	38	-49	65	37	
3.22	...	39	20	-41	75	32	
3.23	Right Cuneus	19	38	-16	92	26	
3.24	...	19	35	-31	83	30	
3.25	...	19	32	-3	91	28	
3.26	...	18	17	-22	97	7	
3.27	Right Inferior Parietal Lobule	40	33	-42	46	56	
3.28	...	39	27	-42	70	42	
3.29	...	40	25	-40	57	54	
3.30	...	40	23	-32	55	59	
3.31	...	40	19	-50	38	51	
3.32	...	40	18	-39	59	47	
3.33	...	40	16	-53	51	44	
3.34	Right Middle Occipital Gyrus	19	39	-39	87	9	
3.35	...	18	24	-26	93	15	
3.36	...	18	21	-32	92	2	
3.37	Right Precuneus	7	35	-25	74	48	
3.38	...	7	27	-11	61	61	
3.39	...	7	26	-13	74	51	
3.40	...	7	23	-2	68	53	
3.41	...	7	22	-1	77	45	
3.42	...	7	19	-7	83	42	
3.43	...	7	18	-13	78	43	
3.44	Right Superior Parietal Lobule	7	32	-24	67	57	
3.45	...	7	28	-35	68	49	
3.46	...	7	25	-23	60	62	

Target network: Smith et al. 7 (auditory - continued).

	Cluster Subdivisions	BA	Voxels	Location			Peak
				x	y	z	t
3.47	Right Supramarginal Gyrus	40	25	-55	56	35	
**4**	**Right Occipital Cluster**	17	54	-6	100	-12	-4.81
4.1	Right Cuneus	17	28	-4	101	-7	
4.2	Right Lingual Gyrus	18	26	-9	101	-18	
**5**	Left Declive		9	45	81	-32	-3.70
**6**	Left Inferior Occipital Gyrus	18	33	33	99	-12	3.95
**7**	Left Inferior Temporal Gyrus		5	70	17	-22	-3.13
**8**	Left Middle Frontal Gyrus		39	32	-68	-7	3.91
**9**	...	8	11	38	-35	46	3.79
**10**	...	10	9	38	-46	31	3.29
**11**	Left Superior Frontal Gyrus	8	6	37	-20	57	2.83
**12**	Left Tuber		6	50	70	-34	-3.41
**13**	Right Middle Frontal Gyrus	10	6	-35	-61	6	-2.66
**14**	Right Postcentral Gyrus	5	21	-17	44	69	4.29
**15**	Right Superior Frontal Gyrus		5	-22	-76	-3	3.29
**16**	Right Tuber		39	-46	79	-35	-4.56

Table S8: Target network: Smith et al. 8 (executive control).

	Cluster Subdivisions	BA	Voxels	Location			Peak
				x	y	z	t
**1**	**Bilateral Frontal Cluster**	10	1206	-10	-40	28	10.36
1.1	Left Medial Frontal Gyrus	10	40	7	-66	10	
1.2	...	10	24	0	-66	-1	
1.3	...	9	17	1	-53	43	
1.4	Left Middle Frontal Gyrus	10	34	33	-44	29	
1.5	...	9	34	26	-43	40	
1.6	...	10	34	30	-55	22	
1.7	...	6	29	20	-17	61	
1.8	...	10	25	32	-60	12	
1.9	...	46	25	42	-46	13	
1.10	...	10	23	40	-54	6	
1.11	...	8	20	25	-34	46	
1.12	Left Superior Frontal Gyrus	10	40	25	-65	1	
1.13	...	6	40	9	-8	69	
1.14	...	10	38	19	-55	30	
1.15	...	9	25	35	-34	34	
1.16	...	10	24	2	-61	32	
1.17	...	6	15	-1	-22	64	
1.18	...	8	12	18	-47	45	
1.19	Right Inferior Frontal Gyrus	45	38	-58	-26	11	
1.20	...	47	26	-58	-26	-4	
1.21	...	10	25	-51	-43	0	
1.22	Right Medial Frontal Gyrus	6	27	-1	-1	70	
1.23	...	10	23	-4	-64	23	
1.24	...	10	17	-8	-66	12	
1.25	Right Middle Frontal Gyrus	46	40	-45	-34	27	
1.26	...	6	38	-21	-6	65	
1.27	...	10	24	-26	-58	26	
1.28	...	10	22	-40	-59	4	
1.29	...	10	19	-47	-54	-5	
1.30	...	10	19	-46	-51	8	
1.31	...	10	18	-46	-46	15	
1.32	Right Precentral Gyrus	44	40	-61	-11	6	
1.33	Right Superior Frontal Gyrus	10	40	-29	-65	8	
1.34	...	9	39	-33	-45	33	
1.35	...	8	37	-8	-42	50	
1.36	...	8	35	-26	-38	47	
1.37	...	6	31	-10	-13	65	
1.38	...	10	30	-37	-54	20	

Target network: Smith et al. 8 (executive control - continued).

	Cluster Subdivisions	BA	Voxels	Location			Peak
				x	y	z	t
1.39	...	6	26	-13	-26	58	
1.40	...	10	24	-22	-51	33	
1.41	...	8	22	-24	-19	56	
1.42	...	9	15	-36	-34	36	
1.43	Right Superior Temporal Gyrus	38	17	-61	-13	-6	
1.44	...	38	15	-57	-22	-14	
**2**	**Left Parietal Cluster**	40	214	42	47	47	-5.99
2.1	Left Inferior Parietal Lobule	40	37	37	54	54	
2.2	...	40	30	47	40	52	
2.3	...	40	24	58	43	44	
2.4	Left Postcentral Gyrus	2	23	62	27	36	
2.5	Left Precentral Gyrus	4	30	57	20	41	
2.6	Left Superior Parietal Lobule	7	26	26	71	48	
2.7	...	7	22	34	65	48	
2.8	...	7	22	19	70	57	
**3**	**Left Temporal Cluster**	21	204	58	59	7	-7.70
3.1	Left Middle Temporal Gyrus	21	40	61	56	2	
3.2	...	21	35	64	41	-1	
3.3	...	19	32	56	64	14	
3.4	...	39	30	52	72	7	
3.5	...	37	28	58	64	-6	
3.6	Left Superior Temporal Gyrus	39	39	56	62	27	
**4**	**Left Occipital Cluster**		171	27	99	0	5.18
4.1	Left Cuneus	18	32	14	109	-12	
4.2	...	18	13	18	107	1	
4.3	Left Inferior Occipital Gyrus	18	36	35	98	-14	
4.4	Left Middle Occipital Gyrus	19	28	37	93	4	
4.5	...	18	25	29	102	-1	
4.6	Left Superior Occipital Gyrus	19	37	29	94	21	
**5**	**Right Temporal Cluster**	21	146	-60	55	1	-6.88
5.1	Right Middle Occipital Gyrus	37	34	-52	72	-1	
5.2	Right Middle Temporal Gyrus	39	37	-58	63	7	
5.3	...	21	34	-65	51	3	
5.4	...	21	26	-68	35	-1	
5.5	...	21	15	-67	44	-5	
**6**	**Right Parietal Cluster**	7	130	-30	56	58	-5.99
6.1	Right Inferior Parietal Lobule	7	30	-37	51	59	
6.2	Right Postcentral Gyrus	3	15	-37	37	62	
6.3	Right Superior Parietal Lobule	7	27	-23	55	64	
6.4	...	7	26	-36	60	55	

Target network: Smith et al. 8 (executive control - continued).

	Cluster Subdivisions	BA	Voxels	Location			Peak
				x	y	z	t
6.5	. . .	7	18	-25	68	54	
6.6	. . .	7	14	-20	66	59	
**7**	**Bilateral Precuneus/Cuneus Cluster**	19	114	-2	82	39	7.82
7.1	Left Cuneus	19	25	6	88	32	
7.2	Left Precuneus	7	29	3	80	42	
7.3	. . .	7	14	12	83	42	
7.4	Right Precuneus	7	27	-9	79	44	
7.5	. . .	19	19	-21	84	38	
**8**	**Left Temporal Cluster**	38	98	59	-14	-3	10.37
8.1	Left Inferior Frontal Gyrus	44	27	60	-15	6	
8.2	Left Superior Temporal Gyrus	38	30	59	-16	-8	
8.3	. . .	22	24	61	-6	2	
8.4	. . .	38	17	56	-22	-15	
**9**	**Right Frontal Cluster**	6	95	-39	12	62	4.83
9.1	Right Middle Frontal Gyrus	6	26	-42	4	59	
9.2	Right Postcentral Gyrus		15	-25	31	78	
9.3	Right Precentral Gyrus	6	29	-35	22	71	
9.4	. . .	6	25	-51	0	49	
**10**	**Left Precentral Gyrus**	6	47	29	27	75	4.03
10.1	. . .	6	27	41	20	65	
10.2	. . .	4	20	29	27	75	
**11**	Left Cuneus	18	8	-1	98	-2	-3.67
**12**	Left Inferior Frontal Gyrus	45	7	54	-36	11	-4.01
**13**	Left Medial Frontal Gyrus	6	6	0	16	77	2.99
**14**	Left Middle Frontal Gyrus	6	9	41	4	59	3.92
**15**	Left Paracentral Lobule	6	13	-2	37	68	-4.23
**16**	Left Postcentral Gyrus	5	9	25	50	73	3.28
**17**	. . .	40	6	69	21	14	3.58
**18**	Left Precentral Gyrus	3	13	13	31	71	-3.66
**19**	. . .	6	5	28	14	63	-3.50
**20**	Left Superior Frontal Gyrus	8	8	33	-19	57	-3.29
**21**	Left Tuber		20	48	76	-37	5.19
**22**	. . .		6	42	85	-35	3.24
**23**	Right Cuneus	18	7	-14	98	4	-3.42
**24**	Right Fusiform Gyrus	18	9	-26	98	-20	4.21
**25**	Right Inferior Parietal Lobule	40	22	-57	44	51	4.40
**26**	. . .	40	17	-64	38	28	3.76
**27**	Right Middle Occipital Gyrus	18	6	-33	97	2	3.33
**28**	Right Middle Temporal Gyrus	21	6	-67	23	-15	3.48
**29**	. . .	21	6	-62	4	-8	-3.75

Target network: Smith et al. 8 (executive control - continued).

	Cluster Subdivisions	BA	Voxels	Location			Peak
				x	y	z	t
**30**	Right Postcentral Gyrus	1	5	-55	18	46	-3.35
**31**	Right Precentral Gyrus	6	7	-12	24	71	-3.96
**32**	Right Superior Frontal Gyrus		21	-16	-76	11	-3.64
**33**	Right Tuber		16	-38	88	-39	4.49
**34**	...		9	-46	80	-37	3.69
**35**	...		8	-52	71	-40	4.33

Table S9: Target network: Smith et al. 9 (frontoparietal).

	Cluster Subdivisions	BA	Voxels	Location			Peak
				x	y	z	t
**1**	**Bilateral Cluster**	40	6410	-15	16	31	28.08
1.1	Left Cuneus	19	34	12	90	27	
1.2	…	19	32	23	91	25	
1.3	…	19	28	30	88	32	
1.4	…	19	26	1	85	33	
1.5	…	19	25	8	89	35	
1.6	…	18	23	13	108	-3	
1.7	Left Fusiform Gyrus	19	24	54	69	-21	
1.8	Left Inferior Occipital Gyrus	18	23	36	95	-14	
1.9	…	18	21	43	91	-7	
1.10	…	18	19	44	85	-14	
1.11	Left Inferior Parietal Lobule	40	40	51	58	42	
1.12	…	39	38	41	71	41	
1.13	…	40	34	58	42	48	
1.14	…	40	32	61	35	41	
1.15	…	40	28	48	50	54	
1.16	…	40	26	43	59	55	
1.17	…	40	25	54	51	45	
1.18	…	39	23	52	67	39	
1.19	…	40	22	59	54	40	
1.20	…	40	19	62	42	32	
1.21	…	40	14	54	42	56	
1.22	Left Lingual Gyrus	18	32	29	101	-9	
1.23	…		18	12	108	-15	
1.24	Left Medial Frontal Gyrus	10	38	7	-67	-2	
1.25	…	10	26	7	-73	7	
1.26	…	10	23	3	-66	8	
1.27	…	9	21	0	-55	46	
1.28	…	8	18	4	-47	49	
1.29	…	10	7	0	-69	17	
1.30	Left Middle Frontal Gyrus	6	38	24	8	66	
1.31	…	6	34	35	3	62	
1.32	…	6	30	26	-8	62	
1.33	…	8	28	31	-34	49	
1.34	…	6	14	19	-19	60	
1.35	Left Middle Occipital Gyrus	18	35	34	96	6	
1.36	…	18	29	24	106	-1	
1.37	…	19	27	58	66	-9	
1.38	…	19	26	38	87	17	

Target network: Smith et al. 9 (frontoparietal - continued).

	Cluster Subdivisions	BA	Voxels	Location			Peak
				x	y	z	t
1.39	...	19	23	53	76	-4	
1.40	...	19	21	48	83	3	
1.41	...	19	20	42	91	3	
1.42	...	19	17	50	78	-14	
1.43	...	18	9	22	98	10	
1.44	Left Middle Temporal Gyrus	39	37	52	74	9	
1.45	...	19	33	48	79	18	
1.46	...	39	22	51	70	28	
1.47	Left Paracentral Lobule	4	26	8	42	69	
1.48	Left Postcentral Gyrus	2	24	36	40	61	
1.49	...	7	23	15	48	66	
1.50	...	2	20	45	33	55	
1.51	...	5	20	25	45	65	
1.52	...	3	20	44	21	59	
1.53	Left Precentral Gyrus	4	29	35	21	63	
1.54	...	4	28	12	33	69	
1.55	...	4	26	24	24	69	
1.56	...	4	22	47	15	54	
1.57	...	4	21	29	31	65	
1.58	...	4	17	16	26	74	
1.59	Left Precuneus	7	38	9	61	58	
1.60	...	19	36	35	80	34	
1.61	...	7	35	17	82	41	
1.62	...	7	31	10	70	51	
1.63	...	7	30	3	78	45	
1.64	...	7	23	-1	61	64	
1.65	...	7	19	23	61	54	
1.66	...	7	15	17	73	52	
1.67	Left Superior Frontal Gyrus		40	0	-63	37	
1.68	...	6	40	6	-28	61	
1.69	...	9	39	25	-46	39	
1.70	...	6	37	5	-7	65	
1.71	...	10	35	27	-69	4	
1.72	...	10	34	23	-65	14	
1.73	...	10	33	15	-67	18	
1.74	...	10	32	21	-69	-4	
1.75	...	10	31	28	-58	27	
1.76	...	6	31	13	16	72	
1.77	...	8	28	10	-53	48	
1.78	...	10	26	14	-64	35	

Target network: Smith et al. 9 (frontoparietal - continued).

	Cluster Subdivisions	BA	Voxels	Location			Peak
				x	y	z	t
1.79	...	8	25	10	-43	56	
1.80	...	9	25	34	-46	35	
1.81	...	10	22	4	-67	27	
1.82	...	8	22	23	-39	48	
1.83	...	10	20	6	-69	21	
1.84	...	6	20	6	7	68	
1.85	...	8	19	17	-46	43	
1.86	...	9	19	21	-56	36	
1.87	...	10	17	12	-61	27	
1.88	...	11	17	4	-68	-13	
1.89	...	6	16	15	8	69	
1.90	...	6	11	15	-9	61	
1.91	Left Superior Occipital Gyrus	19	24	34	93	22	
1.92	...	19	23	43	83	26	
1.93	Left Superior Parietal Lobule	7	34	34	66	50	
1.94	...	7	24	22	75	43	
1.95	...	7	17	33	55	54	
1.96	...	7	15	25	72	49	
1.97	Left Superior Temporal Gyrus	39	31	56	65	19	
1.98	Left Supramarginal Gyrus	40	24	62	50	29	
1.99	Right Cuneus	18	25	-4	89	23	
1.100	...	19	19	-11	87	29	
1.101	Right Declive	19	31	-53	69	-23	
1.102	Right Inferior Frontal Gyrus	46	37	-52	-40	8	
1.103	...	45	36	-56	-21	24	
1.104	...	45	32	-61	-24	9	
1.105	...	47	28	-55	-33	-17	
1.106	...	45	28	-58	-28	17	
1.107	...	9	25	-57	-9	32	
1.108	...	10	25	-47	-52	2	
1.109	...	47	23	-58	-27	-9	
1.110	...	47	23	-60	-19	-3	
1.111	...	45	22	-58	-31	2	
1.112	...	44	21	-62	-13	14	
1.113	...	47	20	-53	-41	-13	
1.114	...	45	17	-60	-10	24	
1.115	Right Inferior Parietal Lobule	40	37	-49	56	52	
1.116	...	40	36	-41	54	54	
1.117	...	39	34	-47	67	41	
1.118	...	40	30	-62	37	42	

Target network: Smith et al. 9 (frontoparietal - continued).

	Cluster Subdivisions	BA	Voxels	Location x	y	z	Peak t
1.119	…	40	30	-67	38	30	
1.120	…	40	27	-66	28	35	
1.121	…	40	25	-56	48	46	
1.122	…	40	24	-52	59	40	
1.123	…	40	22	-61	39	50	
1.124	…	40	20	-51	37	58	
1.125	…	40	19	-51	63	47	
1.126	…	40	16	-55	47	54	
1.127	Right Inferior Temporal Gyrus	20	39	-63	54	-20	
1.128	…	37	32	-61	63	-11	
1.129	…	20	27	-68	19	-26	
1.130	Right Medial Frontal Gyrus	10	38	-16	-69	-1	
1.131	…	6	32	-4	23	71	
1.132	…	6	16	-2	14	69	
1.133	Right Middle Frontal Gyrus	9	40	-55	-19	36	
1.134	…		37	-44	-58	-19	
1.135	…	10	37	-35	-64	11	
1.136	…		35	-52	-49	-17	
1.137	…	10	34	-45	-59	-1	
1.138	…	47	33	-53	-45	-4	
1.139	…	8	33	-45	-15	48	
1.140	…	6	31	-23	-21	61	
1.141	…	8	30	-50	-10	42	
1.142	…	10	30	-41	-63	-11	
1.143	…	6	30	-35	2	61	
1.144	…	9	29	-25	-39	42	
1.145	…	6	29	-25	-7	65	
1.146	…	8	29	-37	-22	49	
1.147	…	8	28	-28	-27	48	
1.148	…	9	28	-39	-30	41	
1.149	…	46	28	-56	-36	20	
1.150	…	9	28	-47	-29	31	
1.151	…	10	27	-46	-47	20	
1.152	…	6	26	-45	-4	51	
1.153	…	9	25	-33	-39	34	
1.154	…	46	24	-48	-50	12	
1.155	…	6	24	-39	-5	60	
1.156	…	46	23	-47	-36	30	
1.157	…	6	22	-52	1	46	
1.158	…	6	22	-34	-10	55	

Target network: Smith et al. 9 (frontoparietal - continued).

	Cluster Subdivisions	BA	Voxels	Location			Peak
				x	y	z	t
1.159	...	10	21	-42	-40	26	
1.160	...	8	14	-47	-26	43	
1.161	Right Middle Temporal Gyrus		38	-63	-2	-32	
1.162	...	39	35	-54	68	13	
1.163	...	21	35	-64	0	-17	
1.164	...	39	31	-53	66	27	
1.165	...	21	30	-69	29	-20	
1.166	...	21	30	-67	48	-8	
1.167	...	21	29	-59	61	-1	
1.168	...	21	28	-65	54	5	
1.169	...	39	28	-49	74	28	
1.170	...	21	25	-69	29	-8	
1.171	...	21	23	-66	15	-10	
1.172	...	21	21	-71	37	-6	
1.173	...	21	19	-66	42	-14	
1.174	...	21	19	-67	13	-18	
1.175	Right Paracentral Lobule	6	28	-1	32	69	
1.176	Right Postcentral Gyrus	43	39	-66	16	17	
1.177	...	2	37	-54	29	52	
1.178	...	4	30	-61	23	44	
1.179	...	5	27	-41	46	62	
1.180	...	3	24	-13	37	69	
1.181	...	7	23	-5	53	66	
1.182	...	2	23	-66	23	26	
1.183	...	3	19	-40	34	63	
1.184	...	5	19	-21	42	66	
1.185	...	3	18	-53	15	53	
1.186	Right Precentral Gyrus	4	33	-42	17	61	
1.187	...	4	28	-56	9	45	
1.188	...	4	27	-62	13	35	
1.189	...	6	26	-60	0	28	
1.190	...	44	26	-61	-11	6	
1.191	...	4	25	-16	25	73	
1.192	...	6	22	-28	21	66	
1.193	...	4	22	-27	31	71	
1.194	Right Precuneus	7	35	-10	74	53	
1.195	...	7	31	-18	83	34	
1.196	...	19	27	-39	74	42	
1.197	...	7	22	-17	69	61	
1.198	...	19	21	-42	77	35	

Target network: Smith et al. 9 (frontoparietal - continued).

	Cluster Subdivisions	BA	Voxels	Location			Peak
				x	y	z	t
1.199	...	7	18	-2	83	43	
1.200	...	7	18	-17	80	44	
1.201	Right Superior Frontal Gyrus	10	39	-30	-66	-3	
1.202	...	10	39	-35	-51	28	
1.203	...	10	38	-16	-62	28	
1.204	...	6	36	-10	-13	65	
1.205	...	8	31	-10	-36	53	
1.206	...	10	30	-28	-59	27	
1.207	...	9	28	-15	-56	45	
1.208	...	8	27	-14	-43	45	
1.209	...	6	27	-17	16	72	
1.210	...	6	26	-10	1	73	
1.211	...	6	26	-20	-14	64	
1.212	...	9	25	-12	-56	36	
1.213	...	6	23	-15	-22	58	
1.214	...	10	23	-24	-64	15	
1.215	...	8	23	-28	-23	57	
1.216	...	8	22	-5	-35	59	
1.217	...	6	20	-2	-24	60	
1.218	...	8	19	-19	-31	49	
1.219	...	6	19	-21	0	69	
1.220	...	10	18	-14	-65	17	
1.221	...	9	17	-29	-48	37	
1.222	Right Superior Occipital Gyrus	19	36	-38	82	29	
1.223	Right Superior Parietal Lobule	7	38	-31	76	46	
1.224	...	7	32	-32	69	55	
1.225	...	7	31	-22	73	54	
1.226	...	7	29	-32	59	62	
1.227	...	7	28	-35	62	52	
1.228	...	7	23	-19	55	65	
1.229	Right Superior Temporal Gyrus	22	35	-68	19	3	
1.230	...	22	35	-60	61	18	
1.231	...	42	34	-67	33	18	
1.232	...	22	34	-67	35	5	
1.233	...	22	27	-64	46	8	
1.234	...	38	26	-59	-15	-30	
1.235	...	22	26	-63	-2	-3	
1.236	...	38	23	-61	-12	-10	
1.237	...	22	14	-65	55	15	
1.238	Right Supramarginal Gyrus	40	36	-60	49	34	

Target network: Smith et al. 9 (frontoparietal - continued).

	Cluster Subdivisions	BA	Voxels	Location x	y	z	Peak t
1.239	…	40	30	-61	57	35	
1.240	…	40	29	-64	48	22	
**2**	**Left Frontal/Central Cluster**	4	254	58	-3	13	-7.70
2.1	Left Inferior Frontal Gyrus	47	39	55	-29	-12	
2.2	…	45	36	54	-28	7	
2.3	…	45	13	58	-10	21	
2.4	Left Postcentral Gyrus	40	20	63	25	22	
2.5	…	42	19	63	19	15	
2.6	Left Precentral Gyrus	6	29	60	0	13	
2.7	…	43	23	61	8	12	
2.8	…	4	23	56	16	41	
2.9	…	4	18	59	13	30	
2.10	…	6	16	60	5	34	
2.11	Left Superior Temporal Gyrus	22	18	59	-11	-1	
**3**	**Left Middle Frontal Gyrus**	9	121	39	-15	49	4.85
3.1	…	9	34	46	-29	34	
3.2	…	9	29	49	-14	38	
3.3	…	9	25	40	-22	43	
3.4	…	46	17	41	-34	27	
3.5	…	8	16	39	-15	49	
**4**	**Left Middle Frontal Gyrus**	11	105	48	-52	-5	5.08
4.1	…	11	35	45	-52	-15	
4.2	…	10	24	41	-61	-8	
4.3	…	10	23	44	-47	14	
4.4	…	47	23	48	-52	-5	
**5**	**Left Temporal Cluster**	21	86	63	9	-23	-5.01
5.1	Left Fusiform Gyrus	20	23	63	7	-32	
5.2	Left Inferior Temporal Gyrus	21	34	64	4	-22	
5.3	…	20	29	64	18	-19	
**6**	**Right Occipital Cluster**	17	83	-13	99	4	5.08
6.1	Right Cuneus	18	32	-18	100	17	
6.2	…	18	25	-8	100	3	
6.3	Right Lingual Gyrus	18	26	-12	99	-11	
**7**	**Left Middle Temporal Gyrus**	21	44	61	51	-1	3.51
7.1	…	21	24	65	39	-8	
7.2	…	21	20	61	51	-1	
**8**	Left Tuber		5	39	88	-38	-2.25
**9**	Right Inferior Occipital Gyrus	18	10	-40	88	-21	2.86
**10**	Right Lingual Gyrus	18	5	-2	104	-18	-2.77
**11**	Right Middle Occipital Gyrus	19	8	-34	89	6	2.89

Target network: Smith et al. 9 (frontoparietal - continued).

	Cluster Subdivisions	BA	Voxels	Location			Peak
				x	y	z	t
**12**	Right Tuber		32	-44	81	-44	-3.70

Table S10: Target network: Smith et al. 10 (frontoparietal).

	Cluster Subdivisions	BA	Voxels	Location			Peak
				x	y	z	t
**1**	**Bilateral Cluster**	40	5266	10	24	28	18.52
1.1	Left Angular Gyrus	39	31	40	78	29	
1.2	...	39	23	49	68	31	
1.3	Left Cuneus	19	34	27	87	30	
1.4	Left Declive	20	31	57	60	-23	
1.5	...		19	50	75	-29	
1.6	Left Inferior Frontal Gyrus	10	36	52	-43	0	
1.7	...	45	36	58	-26	3	
1.8	...	46	33	47	-41	16	
1.9	...	47	30	56	-27	-7	
1.10	...	45	25	54	-22	20	
1.11	...	44	24	59	-11	16	
1.12	...	9	24	58	-19	26	
1.13	...	45	23	53	-31	11	
1.14	...	47	23	55	-41	-10	
1.15	...	47	20	54	-35	-16	
1.16	...	9	19	54	-6	29	
1.17	...		16	50	-44	-21	
1.18	Left Inferior Parietal Lobule	40	39	63	39	28	
1.19	...	40	36	59	34	47	
1.20	...	40	35	56	47	45	
1.21	...	40	35	44	57	44	
1.22	...	40	34	44	44	56	
1.23	...	40	33	49	53	54	
1.24	...	40	31	52	63	40	
1.25	...	40	30	64	27	22	
1.26	...	40	25	62	40	39	
1.27	...	40	20	48	42	49	
1.28	Left Inferior Temporal Gyrus	37	24	62	56	-11	
1.29	...	37	17	58	62	-13	
1.30	Left Medial Frontal Gyrus	8	37	9	-44	47	
1.31	...	10	35	17	-67	0	
1.32	...	6	17	0	13	69	
1.33	...	6	8	13	12	71	
1.34	Left Middle Frontal Gyrus	6	40	31	6	63	
1.35	...	10	40	45	-53	-1	
1.36	...	46	39	42	-38	25	
1.37	...	8	34	45	-10	48	
1.38	...	6	34	21	-5	67	

Target network: Smith et al. 10 (frontoparietal - continued).

	Cluster Subdivisions	BA	Voxels	Location			Peak
				x	y	z	t
1.39	...	6	31	33	-7	60	
1.40	...	8	30	38	-16	46	
1.41	...	10	30	42	-61	-12	
1.42	...	9	30	31	-31	38	
1.43	...	10	29	28	-54	23	
1.44	...	11	27	48	-50	-15	
1.45	...	9	26	55	-6	37	
1.46	...	10	25	29	-63	11	
1.47	...	46	25	47	-46	9	
1.48	...	9	24	56	-19	34	
1.49	...	6	24	45	-1	51	
1.50	...	10	24	37	-54	12	
1.51	...	46	24	55	-32	19	
1.52	...	46	23	45	-29	29	
1.53	...	8	23	27	-29	46	
1.54	...	9	23	43	-25	38	
1.55	...	9	23	48	-18	36	
1.56	...		22	42	-58	-19	
1.57	...	6	20	52	-1	42	
1.58	...	8	15	28	-23	49	
1.59	Left Middle Occipital Gyrus	19	29	52	75	-12	
1.60	...	19	25	58	68	-8	
1.61	Left Middle Temporal Gyrus	39	38	54	68	19	
1.62	...	21	35	65	43	-1	
1.63	...	21	31	65	37	-11	
1.64	...	20	24	64	46	-17	
1.65	...	37	24	60	64	2	
1.66	...	21	23	61	55	0	
1.67	...	39	18	56	69	7	
1.68	...	21	17	65	27	-6	
1.69	...	21	11	66	20	-16	
1.70	Left Paracentral Lobule	4	24	7	39	71	
1.71	Left Postcentral Gyrus	2	36	26	37	71	
1.72	...	5	35	36	49	60	
1.73	...	2	31	51	29	53	
1.74	...	2	24	62	21	29	
1.75	...	2	23	63	27	37	
1.76	...	3	22	44	20	55	
1.77	...	3	20	57	23	41	
1.78	Left Precentral Gyrus	3	32	53	11	45	

Target network: Smith et al. 10 (frontoparietal - continued).

	Cluster Subdivisions	BA	Voxels	Location			Peak
				x	y	z	t
1.79	…	44	32	57	-13	6	
1.80	…	6	31	13	20	72	
1.81	…	4	24	61	5	21	
1.82	…	4	23	58	10	30	
1.83	…	6	23	41	7	55	
1.84	…	4	18	17	32	73	
1.85	Left Precuneus	7	40	18	73	44	
1.86	…	7	40	16	77	52	
1.87	…	39	39	44	73	38	
1.88	…	19	25	34	82	40	
1.89	…	7	24	3	77	41	
1.90	…	7	24	24	62	52	
1.91	…	19	24	26	80	38	
1.92	…	7	21	10	62	55	
1.93	…	7	19	3	77	50	
1.94	Left Pyramis		22	45	80	-42	
1.95	Left Superior Frontal Gyrus	6	39	7	-25	58	
1.96	…	6	35	26	-19	58	
1.97	…	10	33	26	-65	-8	
1.98	…	9	32	19	-48	37	
1.99	…	6	32	6	-2	72	
1.100	…	10	31	34	-62	0	
1.101	…	6	31	10	-9	66	
1.102	…	10	28	17	-58	28	
1.103	…	8	25	19	-36	48	
1.104	…	10	24	12	-64	19	
1.105	…	6	22	19	-17	64	
1.106	…	6	21	10	-16	62	
1.107	…	8	20	32	-13	54	
1.108	…	9	18	9	-53	37	
1.109	…	8	16	20	-31	55	
1.110	…	9	15	29	-41	36	
1.111	…	8	11	2	-37	52	
1.112	Left Superior Occipital Gyrus	19	27	37	86	21	
1.113	Left Superior Parietal Lobule	7	36	32	63	50	
1.114	…	7	35	33	73	49	
1.115	…	7	29	42	62	52	
1.116	…	7	23	8	69	60	
1.117	…	7	23	24	71	57	
1.118	…	7	21	22	62	61	

Target network: Smith et al. 10 (frontoparietal - continued).

	Cluster Subdivisions	BA	Voxels	Location x	y	z	Peak t
1.119	...	7	13	26	55	64	
1.120	Left Superior Temporal Gyrus	38	32	58	-18	-13	
1.121	...	22	30	65	19	6	
1.122	...	22	26	61	3	0	
1.123	...	22	25	59	56	10	
1.124	...	22	21	65	35	8	
1.125	...	39	20	56	63	26	
1.126	...	6	17	63	0	8	
1.127	Left Supramarginal Gyrus	40	32	58	52	36	
1.128	...	40	28	60	52	24	
1.129	...	40	11	57	63	32	
1.130	Left Tuber		3	39	89	-41	
1.131	Right Angular Gyrus	39	19	-56	61	34	
1.132	Right Cuneus	19	33	-17	90	34	
1.133	...	19	14	-30	88	24	
1.134	...	19	11	-16	91	27	
1.135	Right Inferior Frontal Gyrus	47	34	-57	-27	-10	
1.136	...	47	20	-55	-40	-17	
1.137	Right Inferior Occipital Gyrus	18	32	-40	90	-6	
1.138	Right Inferior Parietal Lobule	40	31	-65	35	28	
1.139	...	40	28	-44	48	56	
1.140	...	40	27	-61	34	45	
1.141	...	40	26	-58	48	46	
1.142	...	40	25	-45	56	46	
1.143	...	7	24	-37	64	45	
1.144	...	7	19	-40	71	47	
1.145	...	40	19	-40	58	53	
1.146	Right Inferior Temporal Gyrus	21	33	-67	16	-19	
1.147	...	20	28	-66	7	-28	
1.148	...	20	22	-68	26	-23	
1.149	Right Middle Occipital Gyrus	19	36	-46	84	5	
1.150	...	18	26	-49	80	-14	
1.151	...	19	25	-33	95	2	
1.152	...	19	23	-47	81	-5	
1.153	...	19	22	-33	91	11	
1.154	...	39	19	-46	79	12	
1.155	Right Middle Temporal Gyrus	19	34	-42	82	23	
1.156	...	39	32	-53	72	8	
1.157	...	21	31	-68	29	-9	
1.158	...	39	30	-54	68	23	

Target network: Smith et al. 10 (frontoparietal - continued).

	Cluster Subdivisions	BA	Voxels	Location			Peak
				x	y	z	t
1.159	...	21	30	-61	59	5	
1.160	...	21	29	-65	1	-17	
1.161	...	37	26	-57	68	0	
1.162	...	21	24	-67	7	-7	
1.163	...	21	22	-62	-8	-27	
1.164	...	39	21	-52	73	18	
1.165	...	21	20	-61	-6	-37	
1.166	Right Paracentral Lobule	6	30	-3	34	70	
1.167	Right Postcentral Gyrus	4	37	-52	13	51	
1.168	...	40	35	-55	31	51	
1.169	...	3	30	-18	40	70	
1.170	...	5	27	-33	45	64	
1.171	...	5	25	-24	48	69	
1.172	...	1	24	-64	22	37	
1.173	Right Precentral Gyrus	4	32	-39	31	63	
1.174	...	4	32	-21	29	72	
1.175	...	6	27	-24	14	69	
1.176	...	6	24	-10	21	71	
1.177	...	6	12	-31	25	69	
1.178	Right Precuneus	39	38	-42	73	35	
1.179	...	7	37	-12	52	65	
1.180	...	7	31	-16	80	46	
1.181	...	19	30	-6	85	38	
1.182	...	7	28	-7	70	52	
1.183	...	19	21	-23	84	35	
1.184	Right Superior Frontal Gyrus	6	24	-12	9	70	
1.185	Right Superior Parietal Lobule	7	31	-28	71	46	
1.186	...	7	27	-31	63	55	
1.187	...	7	14	-24	64	58	
1.188	Right Superior Temporal Gyrus	22	40	-67	33	7	
1.189	...	22	33	-66	38	15	
1.190	...	22	31	-65	3	5	
1.191	...	22	29	-64	50	11	
1.192	...	22	29	-67	19	-1	
1.193	...	39	26	-59	59	24	
1.194	...	22	25	-62	-7	-3	
1.195	...	38	23	-60	-13	-11	
1.196	Right Supramarginal Gyrus	22	36	-64	52	21	
1.197	...	40	19	-62	49	37	
1.198	Right Transverse Temporal Gyrus	42	40	-66	19	13	

Target network: Smith et al. 10 (frontoparietal - continued).

	Cluster Subdivisions	BA	Voxels	Location			Peak
				x	y	z	t
**2**	**Right Frontal Cluster**	9	489	-18	-52	33	-6.62
2.1	Right Medial Frontal Gyrus	10	33	-10	-67	8	
2.2	...	10	33	-6	-66	24	
2.3	...	10	23	-3	-67	-3	
2.4	Right Middle Frontal Gyrus	9	36	-28	-40	41	
2.5	...	8	34	-26	-30	51	
2.6	Right Superior Frontal Gyrus	8	37	-16	-41	52	
2.7	...	10	35	-27	-58	28	
2.8	...	9	33	-12	-54	43	
2.9	...	6	32	-14	-29	60	
2.10	...	10	27	-12	-63	33	
2.11	...	10	26	-28	-64	17	
2.12	...	10	25	-20	-70	17	
2.13	...	9	25	-24	-53	38	
2.14	...	9	24	-37	-45	38	
2.15	...	10	22	-10	-71	19	
2.16	...	10	17	-35	-56	20	
2.17	...	8	15	-23	-21	57	
2.18	...	8	12	-6	-47	52	
**3**	**Right Frontal Cluster**	46	322	-50	-27	23	10.07
3.1	Right Inferior Frontal Gyrus	9	40	-56	-11	28	
3.2	...	46	37	-51	-46	8	
3.3	...	46	36	-53	-37	12	
3.4	...	45	33	-56	-22	13	
3.5	Right Middle Frontal Gyrus	46	37	-53	-23	31	
3.6	...	10	31	-48	-52	-8	
3.7	...	6	31	-42	-8	51	
3.8	...	46	27	-53	-34	25	
3.9	...	6	26	-36	-4	61	
3.10	...	46	24	-48	-41	22	
**4**	**Left Lingual Gyrus**	18	59	20	104	-15	3.68
4.1	...	18	39	7	105	-12	
4.2	...	17	20	20	104	-15	
**5**	**Right Inferior Temporal Gyrus**	20	55	-64	51	-11	5.63
5.1	...	20	28	-59	59	-18	
5.2	...	37	27	-64	51	-11	
**6**	**Left Middle Occipital Gyrus**	18	48	30	97	5	-3.49
6.1	...	19	25	47	83	7	
6.2	...	18	23	30	97	5	
**7**	**Left Cuneus**	19	48	11	95	23	-3.86

Target network: Smith et al. 10 (frontoparietal - continued).

	Cluster Subdivisions	BA	Voxels	Location			Peak
				x	y	z	t
7.1	...	19	27	12	93	35	
7.2	...	19	21	11	95	23	
**8**	**Right Cerebellar Cluster**		41	-44	79	-41	4.65
8.1	Right Pyramis		23	-41	84	-44	
8.2	Right Tuber		18	-48	74	-37	
**9**	Left Cuneus	18	5	1	108	4	2.98
**10**	...	18	5	3	88	22	-2.57
**11**	Left Superior Temporal Gyrus		6	54	-24	-36	3.51
**12**	Right Cuneus		5	-17	110	-3	-2.71
**13**	Right Inferior Occipital Gyrus	17	7	-22	100	-17	3.18
**14**	Right Middle Frontal Gyrus		7	-39	-67	-18	2.74

Table S11: Target region: Amygdala.

	Cluster Subdivisions	BA	Voxels	Location			Peak
				x	y	z	t
**1**	**Left Temporal Cluster**	38	67	60	-2	-2	7.19
1.1	Left Middle Temporal Gyrus	21	17	63	8	-8	
1.2	Left Superior Temporal Gyrus	22	25	59	-11	-7	
1.3	...	22	25	61	-2	5	
**2**	**Right Superior Temporal Gyrus**	22	55	-63	2	-2	6.75
2.1	...	22	32	-61	-11	-4	
2.2	...	22	23	-63	2	-2	
**3**	Left Inferior Frontal Gyrus	47	6	54	-28	-12	5.35
**4**	Left Medial Frontal Gyrus	10	20	2	-69	9	4.53
**5**	Left Precentral Gyrus	6	6	47	9	54	5.24
**6**	Right Inferior Frontal Gyrus	47	16	-53	-41	-13	4.88
**7**	Right Superior Temporal Gyrus	42	6	-67	29	18	4.53

Table S12: Target region: Anterior Cingulate Cortex.

	Cluster Subdivisions	BA	Voxels	Location			Peak
				x	y	z	t
**1**	**Left Frontal Cluster**	10	87	30	-50	24	6.94
1.1	Left Middle Frontal Gyrus	10	24	39	-53	12	
1.2	Left Superior Frontal Gyrus	9	37	25	-44	35	
1.3	...	10	26	29	-57	20	
**2**	**Right Frontal Cluster**	10	62	-34	-52	21	6.86
2.1	Right Middle Frontal Gyrus	10	26	-36	-47	25	
2.2	Right Superior Frontal Gyrus	10	36	-33	-57	20	
**3**	Left Medial Frontal Gyrus	10	33	3	-65	14	4.72
**4**	Left Middle Temporal Gyrus	39	33	54	68	6	-4.70
**5**	Left Postcentral Gyrus	3	5	62	17	34	-4.31
**6**	Left Superior Frontal Gyrus	8	11	29	-15	59	-5.15
**7**	Left Superior Temporal Gyrus	38	30	57	-20	-8	6.90
**8**	Right Inferior Frontal Gyrus	47	36	-57	-24	-7	7.81
**9**	Right Inferior Parietal Lobule	40	5	-52	57	42	3.95
**10**	Right Middle Frontal Gyrus	6	7	-31	7	66	-4.35
**11**	Right Middle Temporal Gyrus	37	25	-62	58	1	-5.26
**12**	...	21	5	-66	24	-15	4.09
**13**	Right Precentral Gyrus	4	6	-37	24	71	4.09

Table S13: Target region: Caudate Nucleus.

	Cluster Subdivisions	BA	Voxels	Location			Peak
				x	y	z	t
**1**	Left Inferior Frontal Gyrus	45	23	59	-17	22	4.99
**2**	Left Inferior Occipital Gyrus	18	5	36	96	-22	4.11
**3**	Left Medial Frontal Gyrus	10	37	1	-60	33	5.11
**4**	...	10	7	1	-70	11	4.50
**5**	Left Middle Frontal Gyrus		5	50	-50	-24	4.19
**6**	Left Postcentral Gyrus	5	6	41	48	63	5.35
**7**	Left Superior Frontal Gyrus	10	8	31	-68	4	4.82
**8**	Left Superior Occipital Gyrus	19	18	26	96	23	5.02
**9**	Left Superior Temporal Gyrus	38	22	59	-19	-9	6.30
**10**	Left Supramarginal Gyrus	40	18	62	52	21	-4.19
**11**	Right Inferior Frontal Gyrus	45	16	-60	-22	26	4.76
**12**	Right Lingual Gyrus	18	6	-13	103	-11	4.04
**13**	Right Middle Frontal Gyrus	10	6	-36	-63	17	3.92
**14**	Right Superior Frontal Gyrus	10	12	-35	-68	1	5.43
**15**	Right Tuber		6	-41	87	-37	4.41

Table S14: Target region: Insula Lobe.

	Cluster Subdivisions	BA	Voxels	Location			Peak
				x	y	z	t
**1**	**Right Hemisphere Cluster**	22	489	-59	-8	7	16.28
1.1	Right Inferior Frontal Gyrus	45	34	-61	-11	20	
1.2	…	47	29	-57	-24	-8	
1.3	…	45	29	-53	-38	2	
1.4	…	46	26	-49	-49	3	
1.5	…	47	25	-61	-18	-1	
1.6	…	45	20	-57	-26	9	
1.7	Right Inferior Parietal Lobule	40	37	-65	38	26	
1.8	Right Middle Frontal Gyrus	10	25	-41	-64	-10	
1.9	…	47	22	-50	-51	-7	
1.10	…	10	17	-44	-50	13	
1.11	Right Postcentral Gyrus	40	38	-66	20	15	
1.12	…	2	33	-65	23	28	
1.13	Right Precentral Gyrus	44	31	-62	-10	9	
1.14	Right Superior Temporal Gyrus	22	38	-62	-4	-3	
1.15	…	22	32	-65	5	10	
1.16	…	22	30	-66	35	13	
1.17	…	22	23	-65	12	3	
**2**	**Left Hemisphere Cluster**	22	417	58	-2	9	12.38
2.1	Left Inferior Frontal Gyrus	46	36	47	-45	4	
2.2	…	47	21	55	-26	-4	
2.3	…	44	16	60	-8	18	
2.4	…	45	10	54	-27	7	
2.5	Left Inferior Parietal Lobule	40	33	62	45	25	
2.6	Left Middle Frontal Gyrus	10	36	41	-59	2	
2.7	Left Postcentral Gyrus	40	39	64	25	22	
2.8	Left Precentral Gyrus	44	35	60	-14	9	
2.9	…	6	26	62	-3	8	
2.10	…	6	22	59	1	21	
2.11	Left Superior Temporal Gyrus	22	37	60	-13	-5	
2.12	…	22	32	64	38	13	
2.13	…	22	30	61	4	-1	
2.14	…	22	17	63	20	4	
2.15	Left Transverse Temporal Gyrus	42	27	65	14	13	
**3**	Left Cuneus	18	34	22	107	-8	4.42
**4**	Left Fusiform Gyrus		5	28	100	-22	3.24
**5**	Left Inferior Occipital Gyrus	18	13	40	92	-10	4.66
**6**	Left Middle Frontal Gyrus	6	6	33	4	63	-3.69
**7**	Left Precuneus	19	5	29	84	34	-4.16

Target region: Insula Lobe (continued).

	Cluster Subdivisions	BA	Voxels	Location			Peak
				x	y	z	t
**8**	Left Superior Frontal Gyrus	6	8	12	-16	63	-4.03
**9**	…	10	5	26	-68	1	4.45
**10**	…	10	5	20	-73	14	4.14
**11**	Right Inferior Parietal Lobule	40	10	-43	54	52	-4.34
**12**	Right Medial Frontal Gyrus	6	14	-5	11	70	4.45
**13**	…		6	-9	-58	48	3.81
**14**	Right Middle Frontal Gyrus	9	8	-48	-12	41	-4.43
**15**	…	8	8	-40	-18	47	-3.75
**16**	…	8	5	-29	-26	51	-3.56
**17**	Right Precentral Gyrus	4	6	-56	12	51	4.28
**18**	Right Superior Frontal Gyrus	8	5	-21	-34	46	-3.28
**19**	…	6	5	-4	-23	66	3.76
**20**	Right Tuber		6	-44	83	-38	3.52

Table S15: Target region: Posterior Cingulate Cortex.

	Cluster Subdivisions	BA	Voxels	Location			Peak
				x	y	z	t
**1**	**Left Parietal/Temporal Cluster**	39	192	48	66	30	7.96
1.1	Left Angular Gyrus	39	26	46	75	30	
1.2	Left Inferior Parietal Lobule	7	29	40	67	46	
1.3	Left Middle Temporal Gyrus	39	27	53	71	22	
1.4	Left Precuneus	19	34	39	75	38	
1.5	Left Superior Temporal Gyrus	22	27	58	59	16	
1.6	Left Supramarginal Gyrus	40	30	58	52	31	
1.7	...	39	19	55	62	29	
**2**	**Right Parietal/Temporal Cluster**	39	149	-53	60	29	8.66
2.1	Right Angular Gyrus	39	26	-53	66	31	
2.2	...	39	16	-47	72	33	
2.3	Right Inferior Parietal Lobule	39	21	-43	68	41	
2.4	Right Superior Temporal Gyrus	22	25	-59	58	14	
2.5	Right Supramarginal Gyrus	40	32	-56	54	36	
2.6	...	40	29	-59	53	25	
**3**	**Bilateral Frontal Cluster**	10	81	4	-68	4	5.35
3.1	Left Medial Frontal Gyrus	10	37	12	-69	8	
3.2	Left Superior Frontal Gyrus	10	20	1	-67	-7	
3.3	Right Medial Frontal Gyrus	10	24	-4	-69	7	
**4**	**Bilateral Precuneus Cluster**	7	77	-1	71	50	7.66
4.1	Left Precuneus	7	28	2	60	61	
4.2	Right Precuneus	7	27	-4	75	48	
4.3	...	7	22	-1	80	39	
**5**	**Left Temporal Cluster**	21	60	64	12	-18	6.26
5.1	Left Inferior Temporal Gyrus	21	36	64	5	-23	
5.2	Left Middle Temporal Gyrus	21	24	65	23	-13	
**6**	Left Cuneus		7	12	109	-7	3.85
**7**	...	19	6	8	90	35	-3.92
**8**	Left Inferior Occipital Gyrus	18	5	40	96	-13	3.25
**9**	Left Middle Frontal Gyrus	47	21	47	-49	-3	-5.05
**10**	...	9	10	52	-26	34	-4.37
**11**	...	10	5	40	-48	22	-4.00
**12**	...	6	5	31	5	60	-3.77
**13**	Left Middle Occipital Gyrus		8	33	100	-4	3.99
**14**	Left Postcentral Gyrus		6	15	45	73	4.23
**15**	Left Superior Frontal Gyrus	9	13	24	-42	45	5.05
**16**	Left Superior Occipital Gyrus	19	5	32	95	22	-3.87
**17**	Right Cuneus	19	15	-12	91	35	-4.80
**18**	...	18	10	-14	101	3	4.02

Target region: Posterior Cingulate Cortex (continued).

	Cluster Subdivisions	BA	Voxels	Location			Peak
				x	y	z	t
**19**	Right Inferior Temporal Gyrus	21	16	-67	8	-19	6.22
**20**	Right Middle Temporal Gyrus	21	6	-62	-4	-24	4.13
**21**	Right Superior Frontal Gyrus	10	8	-24	-69	-1	4.00
**22**	...	9	8	-44	-38	37	-4.57
**23**	...	8	7	-25	-37	46	4.49

Table S16: Target region: Putamen.

	Cluster Subdivisions	BA	Voxels	Location			Peak
				x	y	z	t
**1**	**Bilateral Medial Frontal Cluster**	10	88	1	-61	29	6.38
1.1	Left Medial Frontal Gyrus	10	22	0	-68	11	
1.2	Left Superior Frontal Gyrus	9	36	6	-53	45	
1.3	Right Medial Frontal Gyrus	10	30	-3	-66	25	
**2**	**Left Frontal/Temporal Cluster**	45	80	59	-9	8	7.24
2.1	Left Inferior Frontal Gyrus	44	38	60	-6	16	
2.2	...	45	14	58	-19	6	
2.3	Left Superior Temporal Gyrus	22	28	60	-9	-1	
**3**	**Right Frontal/Temporal Cluster**	22	73	-60	-10	6	8.15
3.1	Right Precentral Gyrus	44	26	-62	-6	6	
3.2	...	44	25	-60	-10	15	
3.3	Right Superior Temporal Gyrus	22	22	-59	-16	-2	
**4**	Left Inferior Occipital Gyrus	18	12	31	99	-14	4.27
**5**	...	18	10	40	90	-15	4.12
**6**	Left Middle Occipital Gyrus	19	11	54	70	-15	4.23
**7**	Left Postcentral Gyrus	40	9	64	22	18	5.01
**8**	Left Superior Frontal Gyrus	10	5	24	-67	-2	4.33
**9**	Right Middle Frontal Gyrus	10	19	-40	-65	-11	5.29
**10**	...	47	9	-50	-54	-6	4.61
**11**	...	10	6	-37	-50	25	4.48
**12**	Right Superior Frontal Gyrus	6	16	-3	-23	66	4.96
**13**	...	8	6	-25	-41	49	4.17
**14**	Right Superior Temporal Gyrus	22	8	-68	37	15	5.06

REFERENCES

D'Agostino, R. and Pearson, E. S. (1973). Tests for departure from normality. empirical results for the distributions. *Biometrika* 60, 613–622

D'Agostino, R. B. (1971). An omnibus test of normality for moderate and large size samples. *Biometrika* 58, 341–348

Power, J. D., Plitt, M., Kundu, P., Bandettini, P. A., and Martin, A. (2017). Temporal interpolation alters motion in fMRI scans: Magnitudes and consequences for artifact detection. *PloS one* 12, e0182939. doi:https://doi.org/10.1371/journal.pone.0182939
